# Core–Shell Structures of Bioactive Glass Nanoparticles and MIL-100 Framework: Properties and Biomedical Applications

**DOI:** 10.1021/acsbiomaterials.5c01261

**Published:** 2026-01-29

**Authors:** Marzena Fandzloch, Beata Barszcz, Andrada-Ioana Damian-Buda, Joanna Wiśniewska, Katarzyna Roszek, Grzegorz Słowik, Anna Jaromin, Magdalena Zaremba-Czogalla, Muhammad Asim Akhtar, Aldo R. Boccaccini

**Affiliations:** a 215275 Institute of Low Temperature and Structure Research, Polish Academy of Sciences, Okólna 2, 50-422 Wrocław, Poland; b Department of Material Science and Engineering, Institute of Biomaterials, University of Erlangen-Nuremberg, 91058 Erlangen, Germany; c Faculty of Chemistry, 49577 Nicolaus Copernicus University in Toruń, Gagarina 7, 87-100 Toruń, Poland; d Faculty of Biological and Veterinary Sciences, Nicolaus Copernicus University in Toruń, Lwowska 1, 87-100 Toruń, Poland; e Department of Chemical Technology, Institute of Chemical Sciences, Faculty of Chemistry, 339939 Maria Curie-Sklodowska University in Lublin, 3 Maria Curie-Skłodowska Square, 20-031 Lublin, Poland; f Department of Lipids and Liposomes, Faculty of Biotechnology, 49572 University of Wrocław, F. Joliot-Curie 14a, 50-383 Wrocław, Poland

**Keywords:** Bioactive glass, Metal−organic framework, Hybrid material, Biocompatibility, Bioactivity, Antibacterial, X-ray absorption spectroscopy (XAS)

## Abstract

A novel core–shell hybrid material composed of bioactive glass (BG) nanoparticles and the metal–organic framework (MOF) MIL-100­(Fe) (Fe_3_O­(H_2_O)_2_OH­(BTC)_2_·nH_2_O, BTC: 1,3,5-benzenetricarboxylate) was synthesized using a layer-by-layer strategy. The formation of the MIL-100­(Fe) shell on the BG core was directly confirmed by high-resolution transmission electron microscopy, which revealed a continuous MOF layer with an average thickness of 6.1 ± 0.9 nm. Complementary characterization by infrared spectroscopy, X-ray powder diffraction, X-ray photoelectron spectroscopy, N_2_ sorption, and synchrotron-based X-ray absorption spectroscopy (XAS) confirmed the coexistence of MIL-100­(Fe) and BG components and their structural integrity within the hybrid material. Notably, for the first time, a synchrotron-based technique (XAS) was used to characterize the MOF@BG system, providing unique insight into its local coordination environment and structural evolution. The hybrid material demonstrated favorable cytocompatibility in a long-term (21-day) assay on mouse osteoblast precursor cells (MC3T3) and human dermal fibroblasts (HDF). At the same time, it did not induce *ex vivo* hemolysis at concentrations up to 1000 μg/mL. The induction of osteogenic differentiation in MC3T3 cells in the presence of MIL-100­(Fe)@BG was confirmed by early osteogenic markers, including alkaline phosphatase (ALP) activity and alizarin red staining (ARS). Bioactivity studies in Dulbecco’s phosphate-buffered saline (DPBS) and simulated body fluid (SBF) revealed rapid formation of nanohydroxyapatite, beginning within the first hours of incubation. Importantly, under physiological conditions, the MIL-100­(Fe) shell undergoes a controlled structural transformation, yielding highly dispersed nanoscale Fe_2_O_3_ particles. These nanoparticles induce the production of reactive oxygen species (ROS) and contribute to antibacterial activity, thereby inhibiting *E. coli* and *S. aureus* without the need for external antimicrobial agents. The combination of bioactivity, osteogenic potential, hemocompatibility, and intrinsic antibacterial functionality positions MIL-100­(Fe)@BG as a promising multifunctional platform for bone regeneration and infection control.

## Graphical abstract

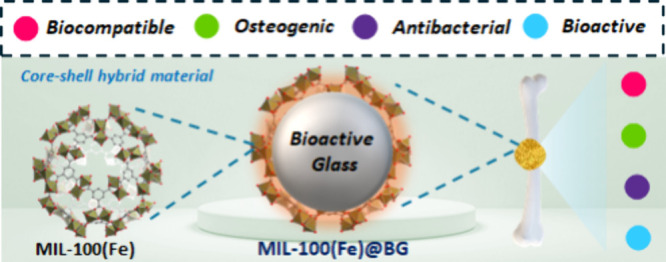

## 1 Introduction

Metal–Organic Frameworks (MOFs) are a promising class of highly porous coordination polymers characterized by exceptional chemical, optical, magnetic, electrochemical, and biological properties. These properties result from the vast range of metal ions or clusters and organic linkers that can be combined to construct MOF architectures.^1^ In particular, the high specific surface area, tunable porosity, structural designability, and remarkable capacity for the encapsulation of gases, ions, nanoparticles, biological compounds, and dyes have enabled applications of MOFs in gas storage and separation, catalysis, optics, luminescence, sensing, and medicine.^2–4^

Among the MOFs investigated for biomedical purposes, iron-based MIL-100­(Fe) (MIL: Materials of Institute Lavoisier) has attracted particular attention. It is one of the few MOFs that have been extensively evaluated for *in vivo* toxicity.^5,6^ MIL-100­(Fe) exhibits a high specific surface area and porosity, a large drug-loading capacity, favorable biocompatibility, and can be readily chemically functionalized. As a result, MIL-100­(Fe) has been widely explored as a drug delivery platform,^7^ supporting controlled release, antimicrobial therapy, and cancer treatment.^8–10^ Furthermore, Hidalgo et al.^11^ demonstrated effective nucleic acid entrapment in the mesopores of nanoscale MIL-100­(Fe), highlighting its potential for gene delivery.

Recently, increasing attention has been focused on the application of MOFs in bone therapy and regeneration.^12^ A broad range of MOF systems, including MOF-5, zeolitic imidazolate framework-8 (ZIF-8), and UiO-66-NH_2_, have been explored for treating bone-related diseases such as tumors, osteoarthritis, osteoporosis, and periodontitis.^13^ Particular interest has been given to the ability of MOFs to release biologically active metal ions (e.g., Zn^2+^, Mg^2+^, Ca^2+^, and Sr^2+^), which enhances osteogenesis and angiogenesis by promoting alkaline phosphatase (ALP) activity, extracellular matrix mineralization, and osteogenic gene expression.^14^ In addition, certain MOFs incorporating transition metal ions have also demonstrated antimicrobial effects, which are of particular importance in preventing implant-associated infection.^15^ MOFs have been used as carriers for osteogenic drugs such as dexamethasone (DEX), as demonstrated in DEX@Zn–Mg–MOF74 ^16^ and SF–DEX@ZIF-8–Ti (SF: silk fibroin) systems.^17^ Another rapidly growing direction is the development of hybrid materials combining MOFs with other biomaterials.^18^

In this context, combining MOF with bioactive glasses (BGs), which play a leading role in advanced bone tissue engineering materials, seems particularly attractive.^19^ A distinguishing feature of BGs compared to other biomaterials is their intrinsic ability to form apatite phases in simulated body fluid (SBF).^20,21^ BGs are already widely used for treatment in various medical fields, including orthopedics, dentistry, and wound healing.^22,23^ Continuous efforts are focused on tailoring their composition, particle size, porosity, and degradation rates to meet specific clinical requirements.^24,25^ In particular, sol–gel-derived BGs have gained recognition due to their high surface reactivity and versatility in producing porous scaffolds, coatings, and intricate structures for regenerative medicine.^23,25,26^ Although multifunctional composite materials combining BG particles and MOFs are still in their infancy, initial studies have demonstrated their promise for biomedical applications.^19,27–29^

Among these examples are mesoporous BG (MBG)/Fe-based MOF three-dimensional (3D) scaffolds, which were investigated for osteoarticular tuberculosis therapy after surgery.^27^ These systems, obtained by simple mixing at different mass ratios of the two components (mMBG:mMOF = 100:0–70:30), were loaded with isoniazid (INH), a first-line antitubercular drug. To address bacterial infection, a major challenge in bone repair, composite MOF@BG systems have also been developed, such as vancomycin-loadedZIF-8 deposited on ternary BG (SiO_2_–CaO–P_2_O_5_) scaffolds (ZIF-8@VAN@BG),^28^ or SiO_2_–CaO substrates coated with NH_4_[Cu_3_(μ_3_–OH)­(μ_3_-4-carboxypyrazolato)_3_] (Cu-MOF) and subsequently decorated with silver nanoparticles.^29^

To the best of our knowledge, previously reported MOF@BG systems were typically obtained by physical mixing or surface decoration and commonly relied on external therapeutic agents such as antibiotics or Ag nanoparticles to achieve antibacterial effects. Such strategies represent a complementary design paradigm to the present work, which focuses instead on a drug-free antibacterial mechanism and on establishing a well-defined nanoscale interface via a layer-by-layer (LBL) core–shell architecture.

Notably, physical mixtures provide poorly controlled MOF/BG interfaces, heterogeneous particle distribution, and limited reproducibility in biological assays. A core–shell architecture offers a fundamentally different strategy: by depositing a conformal MIL-100­(Fe) coating onto BG nanoparticles, a well-defined nanoscale interface is created, enabling (i) more controlled modulation of ion release from BG, (ii) improved reproducibility due to homogeneous surface chemistry, and (iii) a predictable transformation pathway of the MIL-100­(Fe) shell under physiological conditions. This transformation results in the formation of highly dispersed Fe_2_O_3_ nanoparticles, which are expected to contribute to antibacterial activity through ROS-related mechanisms.

Taking this background into account, we report the synthesis of a novel MIL-100­(Fe)@BG hybrid material composed of sol–gel-derived SiO_2_–CaO BG nanoparticles coated with MIL-100­(Fe) using a LBL strategy. Comprehensive physicochemical characterization, including structural evolution under simulated physiological conditions, was performed. The bioactivity, cytocompatibility, osteogenic effects, hemocompatibility, and antibacterial performance of the hybrid were assessed to evaluate its potential as a multifunctional platform for bone regeneration and infection control.

## 2 Experimental Section

### 2.1 Materials and General Methods

All chemicals were commercially available and used without further purification. Calcium hydroxide (≥96%), tetraethoxysilane (TEOS, ≥99%), iron­(III) nitrate nonahydrate (98%), sodium acetate trihydrate (99%), 1,3,5-benzenetricarboxylic acid (H_3_BTC, 95%), sodium chloride (NaCl, ≥99%), dipotassium hydrogen phosphate trihydrate (K_2_HPO_4_·3H_2_O, ≥99%), potassium chloride (KCl, ≥99%), magnesium chloride hexahydrate (MgCl_2_·6H_2_O, ≥99%), and hydrochloric acid (1 M) were purchased from Sigma-Aldrich. Phosphate-buffered saline (PBS), Dulbecco’s phosphate-buffered saline without calcium and magnesium (DPBS), and polyethylene glycol 400 (PEG 400) were obtained from Alfa Aesar. Hydrochloric acid (35–38%), ethanol (96%), and ammonium hydroxide solution (25%) were supplied by Avantor Performance Materials Poland S.A. Iron­(III) oxide (Fe_2_O_3_) and hydrofluoric acid (40%) were purchased from Chempur (Poland). Sodium hydrogen carbonate (NaHCO_3_, ≥99.5%) and tris­(hydroxymethyl)­aminomethane (TRIS, ≥99.5%) were obtained from Carl Roth GmbH (Karlsruhe, Germany), and calcium chloride dihydrate (CaCl_2_·2H_2_O, ≥99%) as well as sodium sulfate (Na_2_SO_4_, ≥99%) were purchased from VVW Chemicals. Ultrapure water (18.2 mΩ cm) was used throughout. SBF was prepared according to the protocol established by Kokubo et al.^30^ [Fe_3_O­(OOCCH_3_)_6_OH]·2H_2_O cluster was synthesized according to a synthesis previously described by Wang et al.^31^ Elemental analysis calcd. (%): C 25.12, H 3.86; found: C 25.33, H 4.16. MIL-100­(Fe) was prepared by a known synthesis under HF-free conditions.^32^

Powder X-ray diffraction (PXRD) patterns of BG nanoparticles, MIL-100­(Fe), and MIL-100­(Fe)@BG were collected on an X’Pert PRO diffractometer (PANalytical) using Cu Kα radiation (λ = 1.5418 Å). Mid-infrared spectra (4000–400 cm^–1^) were recorded with a Thermo Scientific Nicolet Summit X Fourier-transform infrared spectrometer using KBr pellets. X-ray photoelectron spectroscopy (XPS) measurements were carried out using a PREVAC multichamber UHV system. UV–vis diffuse reflectance spectra were obtained on an Agilent CARY 5000 UV–vis-NIR spectrophotometer. Thermogravimetric (TG) analysis and differential thermal analysis (DTA) were performed using a Setaram SETSYS TG-DTA 16/18 equipment, at a heating rate of 10 °C/min in flowing air. Elemental analysis (C, H, N) of the [Fe_3_O­(OOCCH_3_)_6_OH]·2H_2_O cluster was conducted using a CHNS Vario EL Cube Elementar analyzer. Specific surface areas of BG nanoparticles and MIL-100­(Fe)@BG were determined from N_2_ adsorption–desorption isotherms recorded at 77 K using a Micromeritics 3Flex surface characterization analyzer. Prior to isotherm acquisition, the materials were activated and outgassed (150 °C, 1.3 kPa) for 12 h. MicroActive software was used to determine specific surface area according to the Brunauer–Emmett–Teller (BET) method and total pore volume at a relative pressure (*p*/*p*_0_) = 0.9. Transmission electron microscopy (TEM) images were acquired using a TECNAI G2 20 X-TWIN microscope equipped with a LaB_6_ cathode electron gun, FEI Eagle 2K CCD camera, a high-angle annular dark-field imaging (HAADF) detector for the scanning transmission electron technique (STEM), and X-ray microanalyser for the determination of the energy-dispersive X-ray spectroscopy (EDS) elemental composition. Measurements were performed at an accelerating voltage of 200 kV in bright field, dark field and scanning-transmission modes. The particle size distribution for BG was obtained by measuring 100 particles from TEM images using ES Vision software. STEM images of MIL-100@BG_1L and MIL-100@BG_3L were acquired using a Thermo Fisher Scientific SCIOS 2 LoVac FIB-SEM microscope. High-resolution TEM (HRTEM) images with Fast Fourier Transform (FFT) analysis and STEM-EDS elemental mapping were performed using a FEI Titan G2 60–300 microscope. The main equipment of the microscope included a field emission gun (FEG), a monochromator, a three-condenser lens system, an objective lens system, an image Cs-corrector, a HAADF detector, and an EDS spectrometer with a Si­(Li) detector. Microscopic measurements were performed at an accelerating voltage of 300 kV. The MIL-100­(Fe) shell thickness was determined by statistical analysis of 100 measurements obtained from HRTEM images. Zeta potential measurements were carried out using a Zetasizer Nano ZS (Malvern Instruments) for BG and MIL-100­(Fe)@BG dispersed in DPBS or distilled water (1.5 mg/mL), sonicated for 30 s prior to analysis. Each value represents six measurements performed in three independent experiments (average of 100 runs). The composition of powders was determined using an iCAP 7400 ICP-OES analyzer. To quantify the content of elements (Ca, Si, P, Fe) by inductively coupled plasma-optical emission spectrometry (ICP-OES), 1–2 mg of powder was degraded in a mixture of HF/HCl (1 : 1, 6 mL) and subsequently diluted with distilled water (30 mL). Each material was measured in triplicate, originating from three independent syntheses. The reported weight percentage values and standard deviations reflect synthesis reproducibility. Additionally, supernatants collected after incubation in DPBS or SBF at 4, 24, and 72 h (prior to the first medium exchange) were acidified with 1 mL of HCl and analyzed by ICP-OES to monitor Fe release.

### 2.2 X-ray Absorption Studies

X-ray absorption spectra were recorded at the National Synchrotron Radiation Centre SOLARIS in Cracow, Poland, at the bending magnet PEEM/XAS beamline within the energy range of 200–2000 eV, covering the K-edges of O (500–550 eV) and Si (1800–1900 eV), as well as the L-edge of Ca (300–400 eV) and Fe (700–750 eV). The X-ray absorption spectra were performed for the [Fe_3_O­(OOCCH_3_)_6_OH]·2H_2_O cluster, MIL-100­(Fe), BG, and MIL-100­(Fe)@BG, respectively. Samples were finely placed on an experimental double-sided adhesive conductive graphite tape mounted on an Omicron plate (flag style) sample holder made of titanium. The maximum area available on the Omicron plate for mounting samples was 15 mm × 11 mm (H x V). The beam spot size limited the minimum sample size at the end of the X-ray absorption spectroscopy (XAS) station to 0.25 mm × 0.05 mm. To avoid loss of intensity of the radiation beam, the preferred sample size was larger than the size of the beam trace on the sample and was usually about 2–3 × 1 mm (H x V). The synchrotron radiation beam was scattered in several additional experiments to reduce the total radiation power hitting the sample. A step size of 0.1 eV was used for all XAS spectra for the edge regions and 0.2 eV for the remaining areas. Attenuation lengths for photons in the soft X-ray regime typically range from 0.1 to 1 μm in the 400–2000 eV energy range. Above 2000 eV, attenuation lengths can grow up to hundreds of microns, while below 400 eV, they can approach 0.01 μm. The data sets were collected at room temperature under ultrahigh vacuum (UHV) in total electron yield (TEY) mode. The data were processed using the PyMca 5.4.0 software package and evaluated using OriginPro as the data analysis and graphing software.

### 2.3 Synthesis of Materials

#### 2.3.1 Synthesis of BG Nanoparticles

BG nanoparticles were prepared following our previously reported procedure with minor modifications.^25^ Reagent quantities were adjusted to obtain a theoretical oxide composition of 70SiO_2_–30CaO (wt %). For this purpose, three mixtures were prepared: (1) TEOS (2.34 mL) and ethanol (20 mL); (2) distilled water (11.7 mL), ethanol (17.5 mL), and ammonia solution (0.6 mL). After simultaneous stirring of mixtures (1) and (2) for 30 min on a magnetic stirrer, mixture (1) was added dropwise to mixture (2), and the resulting solution was stirred for 2 h. Subsequently, mixture (3), consisting of calcium hydroxide (0.358 g) suspended in polyethylene glycol (10 mL) for 2 h, was added to the reaction mixture and stirred for 24 h at room temperature. The resulting precipitate was collected, washed with distilled water (3 × 30 mL) and ethanol (2 × 30 mL), and dried at 50 °C for 24 h. The dried powder was ground in a mortar and calcined at 650 °C for 3 h, with a heating rate of 5 °C/min (cooling without temperature control). The chemical composition determined by ICP-OES was as follows: Ca = 13.28 ± 1.01 wt % and Si = 23.25 ± 1.15 wt %.

#### 2.3.2 Synthesis of MIL-100­(Fe)@BG

The new hybrid material was prepared according to a LBL method. For this purpose, 0.250 g of BG nanoparticles were suspended in an ethanolic solution of H_3_BTC (10 mL, 2.5 mM) and stirred for 30 min (1000 rpm) at 60 °C. Afterward, the precipitate was centrifuged and washed with ethanol (1 × 10 mL). The obtained material was subsequently immersed in an ethanolic solution of the [Fe_3_O­(OOCCH_3_)_6_OH]·2H_2_O cluster (10 mL, 2.5 mM) and stirred for 15 min (1000 rpm) at 60 °C. The resulting product was centrifuged and washed with ethanol (1 × 10 mL). This deposition cycle was repeated 5 times. Finally, the hybrid material was dried overnight at 50 °C. According to ICP-OES measurements, the MIL-100­(Fe)@BG nanoparticles exhibited the following chemical composition: Fe = 5.57 ± 0.44 wt %, Ca = 11.85 ± 0.84 wt %, Si = 23.60 ± 1.93 wt %.

Intermediate samples with 1 and 3 deposition cycles (MIL-100­(Fe)@BG_1L and MIL-100­(Fe)@BG_3L, respectively) were prepared using the same LBL procedure, with 1 or 3 consecutive deposition cycles instead of 5.

### 2.4 Bioactivity Determination

#### 2.4.1 Evaluation of HA Mineralization

Hydroxyapatite (HA) formation was determined in both DPBS and SBF. To maintain the previously established protocol,^25^ equal amounts of powders (MIL-100­(Fe)@BG or BG) were immersed in DPBS or SBF at a concentration of 1.5 mg/mL in clean and sterile polyvinyl chloride bottles. Afterward, the samples were placed inside an incubator at a controlled temperature of 37 °C under continuous stirring (100 rpm) for 4 h, 24 h, 72 h, 7 d, 14 d, and 21 d. The medium was regularly refreshed after 3.5 days. At each selected time point, the powder was filtered from the solution, rinsed with distilled water (3 × 5 mL), and dried at 60 °C for 24 h. HA mineralization on the surface of BG or MIL-100­(Fe)@BG nanoparticles was determined by PXRD, Fourier transform infrared (FTIR) spectroscopy, and TEM imaging. Changes in the composition of the materials during 21 days of incubation were monitored by ICP-OES. Additionally, pH changes and Fe release into the incubation medium were quantified by ICP-OES at 4, 24, and 72 h, prior to the first media exchange.

#### 2.4.2 Surface Charge Evolution during Incubation in DPBS

Time-dependent changes in the zeta potential of MIL-100­(Fe)@BG were monitored during incubation in DPBS at a concentration of 1.5 mg/mL. Measurements were performed after 5 min, 15 min, 30 min, 1, 2, 4, 6, and 24 h under the same instrumental conditions described previously (Section 2.1).

### 2.5 Stability of MIL-100­(Fe) in DPBS

Following a procedure analogous to the bioactivity test, equal amounts of MIL-100­(Fe) powder were immersed in DPBS at a concentration of 1.5 mg/mL in clean and sterile polyvinyl chloride bottles. The samples were placed in an incubator at 37 °C under continuous stirring (100 rpm) for 4 h, 24 h, 72 h, 7 d, 14 d, and 21 d. In this experiment, no medium exchange was performed. At each selected time point, the powders were recovered by filtration, rinsed with distilled water (3 × 5 mL), and dried at 60 °C for 24 h. Structural changes occurring during incubation were evaluated by PXRD and FTIR. The final sample obtained after 21 d was additionally examined by HRTEM. Fe leaching during the 21-day incubation period was monitored by ICP-OES, and pH changes of the incubation solution were also recorded.

### 2.6 *Ex Vivo* Red Blood Cell Hemolysis Assay

The Bioethics Commission at the Lower Silesian Medical Chamber approved (1/PNHAB/2018) the method used for the determination of hemolysis described by Jaromin et al.^33^ Freshly isolated human erythrocytes (hematocrit of 50%) were gently mixed with BG or MIL-100­(Fe)@BG (final concentration 50, 250, 500, and 1000 μg/mL, respectively) in PBS buffer. All samples were incubated at 37 °C in a water bath for 30 min and then centrifuged at 650 × g for 10 min at room temperature. The absorbance of the supernatants was measured at 540 nm. The following controls were used in the experiment: negative (erythrocytes in PBS buffer) and positive (erythrocytes in double-distilled water), respectively. Each concentration was tested in five replicates.

### 2.7 *In Vitro* Cytotoxicity Assessment

The *in vitro* cytotoxicity was assessed on two types of cells: human dermal fibroblasts (HDF) and mouse osteoblast precursors (MC3T3). HDF cells were purchased from Biokom (Poland). The cells were cultured in DMEM-LG (Dulbecco’s Modified Eagle’s Medium, Low Glucose) medium containing 10% FBS (Fetal Bovine Serum) and 1% penicillin/streptomycin solution, at 37 °C in a humidified atmosphere with 5% CO_2_. MC3T3 cells were purchased from Sigma-Aldrich (Germany) and cultured according to the manufacturer’s instructions in EMEM (Eagle’s Minimum Essential Medium) containing 10% FBS and 1% penicillin/streptomycin solution, at 37 °C in a humidified atmosphere with 5% CO_2_. A volume of 5 μL containing approximately 1 × 10^4^ cells was seeded in each well of the 96-well plate 24 h before the experiments. For cytotoxicity evaluation, the tested materials were added to the growing cells at concentrations ranging from 1 to 1000 μg/mL and incubated for 24 h, 72 h, 144 h, and 21 d. Subsequently, the MTT (3-(4,5-dimethylthiazol-2-yl)-2,5-diphenyltetrazolium bromide) test, which measures the ability of the cells to reduce MTT by mitochondrial dehydrogenases, was performed in triplicate to assess the cell metabolic activity (viability). The formed formazan crystals were dissolved in dimethyl sulfoxide (DMSO) and the absorbance was measured at 570 nm (with a reference wavelength of 630 nm) using a Synergy HT Multidetection reader (BioTek Instruments, Winooski, VT, USA).

### 2.8 Alizarin Red S Staining

Growing MC3T3 cells were treated with the tested materials for 72 h, 144 h and 21 d. Then, the cells were fixed for 15 min in 10% formalin solution. After that time, the residual formaldehyde was removed by washing the wells twice with bidistilled water. Extracellular calcium deposits were stained by incubating the samples for 20 min with 100 μL Alizarin Red solution, having a pH of 4.2 (Sigma-Aldrich, Germany). Unbound dye residues were removed by washing the wells three times for 3 min with distilled water. For quantitative analysis, the stained calcium deposits were dissolved in 100 μL of 10% acetic acid by shaking for 30 min at 37 °C. The absorbance was measured spectrophotometrically at a wavelength of 405 nm. The number of cells determined from the MTT assay for each sample was used to calculate the normalized alizarin red staining (ARS). The assay was performed in triplicate.

### 2.9 Alkaline Phosphatase (ALP) Activity

The first step in determining ALP enzyme activity was to obtain cell lysates of MC3T3 cells treated with the tested materials for 72 h, 144 h and 21 d. First, 100 μL of lysis buffer was added to each well, incubated for 10 min at 37 °C, and the cells were mechanically disintegrated with a scraper. The obtained lysates were centrifuged (3 min, 3000 g) and 50 μL aliquots were transferred to the wells to determine the catalytic activity of the enzyme. The substrate used for the enzymatic reaction was 1 mM *p*-nitrophenylphosphate (pNPP, Thermo Scientific Chemicals, USA). Enzyme activity was determined by adding 50 μL of substrate solution to 50 μL of cell lysate. A blank sample containing 50 μL of lysis buffer and 50 μL of substrate solution was also prepared. All samples were incubated for 90 min at 37 °C and then the reaction was stopped by adding 0.1 mL of 1% NaOH solution. The absorbance of the samples was measured at 405 nm using a Synergy HT Multidetection reader. The number of cells determined in the MTT assay for each sample was used to calculate the normalized activity of ALP. The assay was performed in triplicate.

### 2.10 Antibacterial Test

The antibacterial activity was carried out on *Escherichia coli* (ATCC25922) and *Staphylococcus aureus* (ATCC25923) bacterial strains obtained from the Leibnitz Institute DSMZ-German Collection of Microorganisms and Cell Cultures (Braunschweig, Germany).^34^ Before starting the antibacterial tests, the powders were sterilized by UV irradiation for 2 h. Subsequently, the samples were incubated in lysogeny broth (LB) medium (Carl Roth GmbH, Karlsruhe, Germany) at a concentration of 1.5 mg/mL. During this time, both bacterial strains were cultured in LB medium at 100 rpm and 37 °C in order to obtain a fresh bacterial suspension suitable for inoculation. The following day, the medium was extracted from the powders by centrifugation (4500 rpm, 10 min), while the optical density (OD) of the bacterial suspension, measured at 600 nm (PHOmo, Anthos Mikrosysteme GmbH, Germany), was adjusted to 0.015. Afterward, the bacterial suspension was added to the previously extracted medium, with the bacterial suspension cultured in fresh LB medium serving as the control group. For the blank, plain LB medium was used. At predetermined time points (3, 6, and 24 h), the OD of the bacterial suspensions was quantified at 600 nm and the relative bacterial viability was calculated according to the following equation:

$$Relative\;bacterial\;viability\;\left(\%\right)=\frac{OD_{sample}-OD_{blank}}{OD_{control}-OD_{blank}}\times 100$$

Where *OD*_*sample*_, *OD*_*blank*_ and *OD*_*control*_ are the optical densities of the sample, blank and control, respectively. For each composition, the experiments were performed in triplicate.

### 2.11 Assessment of ROS Generation in *E. coli* Induced by MIL-100­(Fe)@BG

The level of intracellular reactive oxygen species (ROS) was evaluated using bacterial cell staining with the cell-permeable probe 2′,7′-dichlorofluorescein diacetate (DCFH-DA, Sigma-Aldrich, ≥97% (HPLC)). Prior to ROS assessment, the powder samples were prepared following the same procedure as used in the antibacterial test: UV sterilization for 2 h, incubation in LB medium (1.5 mg/mL) overnight (37 °C, 137 rpm), and subsequent centrifugation at 4500 rpm for 10 min to separate the medium. *Escherichia coli* (ATCC 8739) was cultured overnight in LB medium (37 °C, 137 rpm) to obtain fresh bacterial suspensions for inoculation. The following day, the bacterial suspension was added either to fresh LB medium (untreated control) or to the medium extracted from the powder samples incubated as described above. The bacterial cultures were then incubated for 3, 6, and 24 h (37 °C, 137 rpm). After incubation, the suspensions were centrifuged at 300 × g for 30 min at 4 °C. The resulting pellets were washed with PBS and resuspended in PBS containing 100 μM DCFH-DA, followed by incubation in the dark for 1 h. During staining, DCFH-DA is hydrolyzed by intracellular esterases to nonfluorescent 2′,7′-dichlorodihydrofluorescein (DCFH), which is subsequently oxidized by ROS to form the fluorescent compound 2′,7′-dichlorofluorescein (DCF). Fluorescence intensity was measured using a Cary Varian spectrofluorometer at an excitation wavelength of 485 nm and an emission wavelength of 528 nm. The results are based on two independent experiments, each performed in triplicate. The results are expressed as fold change relative to the control group (bacteria incubated with DCFH-DA).

## 3 Results and Discussion

### 3.1 Synthesis and Characterization of BG and MIL-100­(Fe)@BG

BG with a composition of 73SiO_2_–27CaO wt % was prepared through a modified Stöber process following our previously described procedure.^25^ In this sol–gel approach, calcium hydroxide was used as the source of calcium. One modification involved the addition of a different amount of ammonia, which affects the size of the BG particles.^25,35^ This resulted in nanoparticles with a size of 71 ± 10 nm (Figure S1). Through PXRD analysis (Figure 1a), the amorphous nature of the resulting BG nanoparticles was confirmed, exhibiting a characteristic broad signal within the 20–30° 2θ range. Further characterization of the textural properties revealed that BG nanoparticles exhibit a type IV isotherm, which is a characteristic feature of mesoporous materials (Figure 1d). The BET surface area of BG nanoparticles was measured to be 31 m^2^/g.

**Figure 1:**
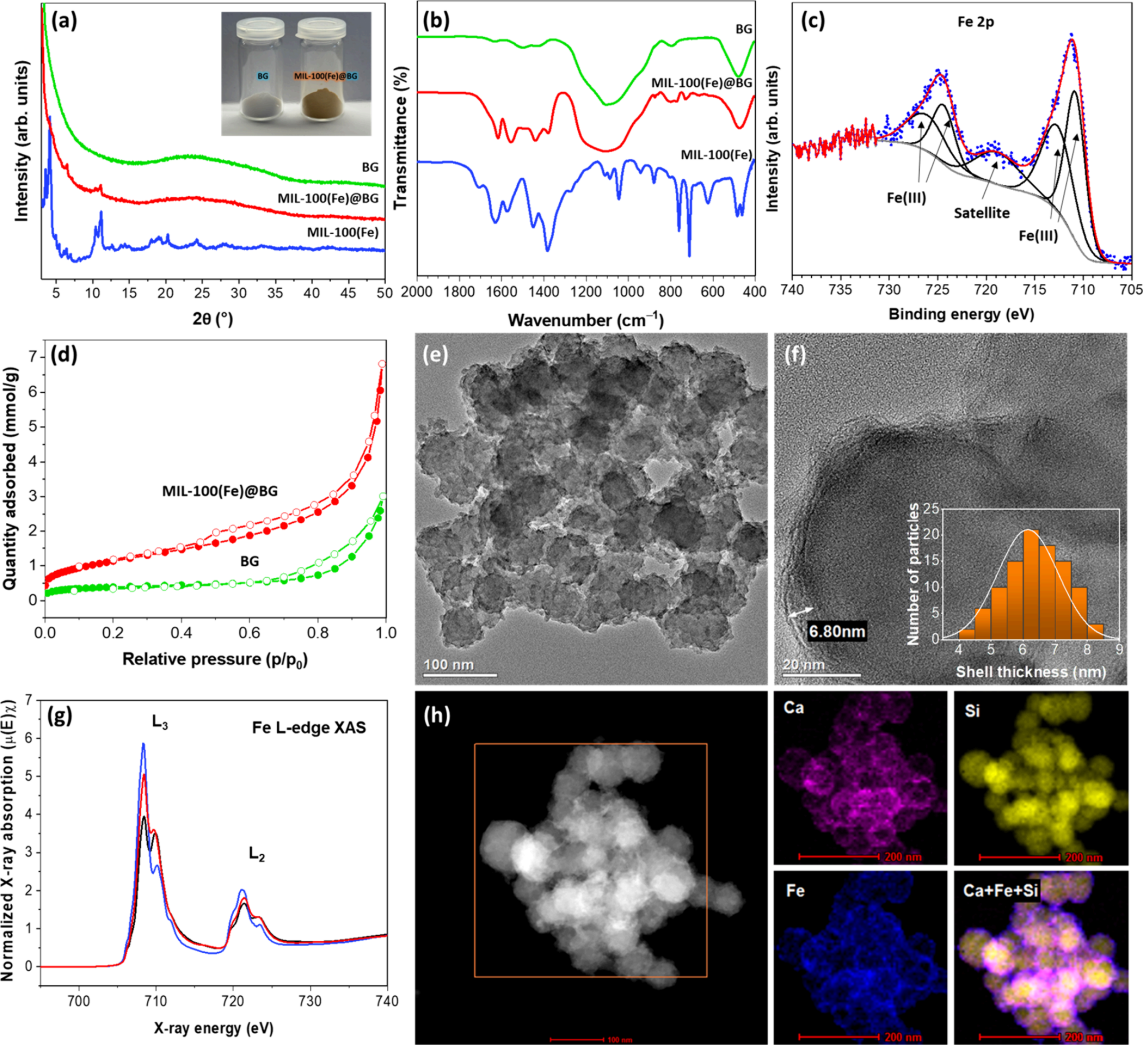
PXRD patterns (a) and FTIR spectra (b) of BG, MIL-100­(Fe)@BG, and MIL-100­(Fe). High-resolution XPS spectrum of Fe 2p for MIL-100­(Fe)@BG (c). N_2_ adsorption (filled symbols) and desorption (empty symbols) isotherms of BG and MIL-100­(Fe)@BG (d). HR-TEM and STEM images with EDS elemental mapping of MIL-100­(Fe)@BG (e, f; inset shows the shell thickness distribution, h). Normalized Fe L-edge XAS spectra of the Fe­(III) cluster (black), MIL-100­(Fe) (blue), and MIL-100­(Fe)@BG (red) (g).

The resulting BG nanoparticles were used in the synthesis of a new hybrid material. MIL-100­(Fe)@BG was synthesized following a LBL approach (Scheme 1). In this strategy, the preformed iron­(III) acetate trinuclear cluster [Fe_3_O­(OOCCH_3_)_6_OH]·2H_2_O was employed instead of the FeCl_3_·6H_2_O precursor. This eliminates the need for *in situ* secondary building unit (SBU) formation and enables faster and more controlled nucleation of MIL-100­(Fe) framework, a behavior that was further verified through characterization of intermediate products obtained after the first and third deposition cycles (denoted MIL-100­(Fe)@BG_1L and MIL-100­(Fe)@BG_3L, respectively). For this purpose, the BG nanoparticles were suspended in an ethanolic solution containing the H_3_BTC ligand and stirred at 60 °C for 30 min. The nanoparticles were then recovered by centrifugation, washed with ethanol, resuspended in an ethanolic solution of the Fe­(III) cluster, and stirred at 60 °C for 15 min. This cycle was repeated five times. The washing process enabled the removal of unbound MIL-100­(Fe) components, thus ensuring the homogeneity of the final hybrid material. With each subsequent layer, the color of the final material changed from white to bright orange, as shown in Figure 1a (inset). Throughout the manuscript, the material obtained after 5 deposition cycles is referred to as MIL-100­(Fe)@BG.

**Scheme 1:**
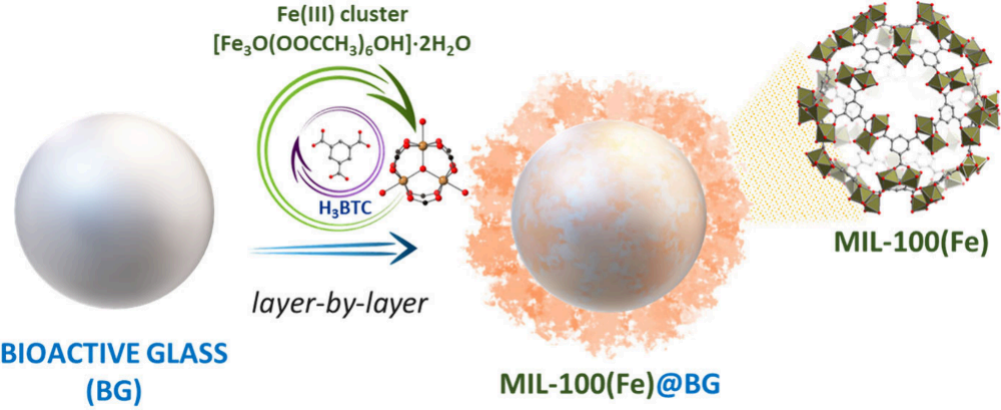
Synthesis of MIL-100­(Fe)@BG Using a Layer-by-Layer (LBL) Strategy

The mechanism of MIL-100­(Fe) formation on BG nanoparticles, which are rich in hydroxyl groups on their surface,^25^ may be similar to that reported by Lee et al. for cotton fabric with −OH groups.^36^ These hydroxyl groups can act as binding sites for MOFs without the need for prior surface treatment. Possible interactions between the silanol groups Si–OH and carboxylic acids have been reported previously.^37,38^ According to Kulik et al.,^37^ the chemisorption of carboxylic acids on the surface of silica occurs via a nucleophilic substitution reaction of the silanol group, leading to the formation of chemically bound carboxylic acid groups and the release of a water molecule. The LBL process was repeated to achieve uniform growth of MOFs on the BG surface, due to the participation of the remaining hydroxyl groups.

Once the hybrid material was isolated, the deposition of MIL-100­(Fe) on the surface of the BG nanoparticles was initially examined by PXRD. The diffraction pattern of MIL-100­(Fe)@BG (Figure 1a) shows low-intensity reflections characteristic of MIL-100­(Fe) at low angles (3–11° 2θ), whereas the broad hump in the 20–30° 2θ range is characteristic of the amorphous BG phase. This behavior is consistent with previously reported hybrids containing a small amount of MIL-100­(Fe) obtained as a colloidal layer. In such cases, only weak and broadened MIL-100­(Fe) diffraction peaks were observed on different substrates, including HA nanorods,^39^ HA nanowires,^40^ cotton fabric,^36^ Au nanorods,^41^ or Fe_3_O_4_ supraparticles.^42^

FTIR spectroscopy clearly confirmed the dual nature of the hybrid material. Characteristic bands corresponding to both BG nanoparticles (at 1098, 797, and 474 cm^–1^, related to stretching and bending vibrations of Si–O–Si)^43^ and MIL-100­(Fe) (at 1617–1379 cm^–1^, attributed to the symmetric and asymmetric motions of the COO^–^ group and C=C stretching vibrations, as well as two bands at 772 and 729 cm^–1^ related to C–H bending vibrations of BTC)^44,45^ were identified in the hybrid material (Figure 1b). For the intermediate samples, MIL-100­(Fe)@BG-1L and MIL-100­(Fe)@BG-3L, FTIR spectra (Figure S2) revealed the progressive development of the MIL-100­(Fe) contribution: after one deposition cycle, the MIL-100­(Fe)-related bands were already detectable, but the BG bands remained dominant, whereas after three cycles the framework bands became substantially more pronounced. This systematic evolution of the spectra from 1L to 3L and 5L provides clear spectroscopic evidence for progressive shell growth with increasing numbers of LBL cycles.

XPS analysis performed on MIL-100­(Fe)@BG confirmed the presence of Ca and Si originating from the BG component. The binding energies of Ca 2p_3/2_ and 2p_1/2_ appeared at 347.4 and 350.9 eV, respectively (Figure S3a). In the Si 2p region, a dominant peak at 103.3 eV was observed, characteristic of network-forming SiO_4_ tetrahedra (Q^4^ species), accompanied by a shoulder at 101.8 eV attributed to Q^3^ species (Figure S3b). This feature may suggest partial depolymerization of the silicate network, likely associated with Si–O–Ca chemical environments in the hybrid material. A shift of the Si 2p signal toward lower energies with increasing alkali metal ion content has been previously reported for bioactive glasses BG50 and BG42, as well as for colloidal silica.^46,47^ The Fe 2p high-resolution spectrum displayed characteristic peaks at 711.1 eV (Fe 2p_3__/__2_) and 724.6 eV (Fe 2p_1__/__2_), with a separation of 13.5 eV, along with a satellite peak at 718.8 eV (Figure 1c), consistent with the presence of Fe­(III) in the MIL-100­(Fe) structure.^48^ Additionally, analysis of the O 1s and C 1s regions (Figure S3c,d, Table S1) showed contributions from both MIL-100­(Fe) and BG, confirming the coexistence of both components in the final hybrid material.

In addition to surface chemical analysis, the optical properties of the new material were assessed using diffuse reflectance spectroscopy (DRS). As shown in Figure S4a, the absorption spectrum of MIL-100­(Fe)@BG exhibited notable differences compared to that of pristine BG, which showed negligible absorption in the 200–800 nm range. In contrast, MIL-100­(Fe) revealed an absorption band in the UV region with an asymmetric tail extending into the visible range (220–600 nm, maximum at about 285 nm), attributed to ligand-to-metal charge transfer (LMCT) from the organic linker to Fe^3+^ centers.^49^ A distinct increase in absorption was also observed for the hybrid material in this region, with a maximum at around 225 nm, primarily reflecting the contribution of MIL-100­(Fe). The optical band gap energy (E_g_) was estimated using the Tauc plot derived from the modified Kubelka–Munk function.^50^ The analysis revealed a band gap of approximately 3.96 eV for MIL-100­(Fe)@BG, whereas the band gap of pristine MIL-100­(Fe) typically ranges from 2.49 to 3.08 eV, depending on synthesis conditions (2.98 eV as determined in this study, Figure S4b).^48,49,51,52^

Due to the presence of the porous MOF component, the textural properties of MIL-100@BG changed significantly compared with BG (Figure 1d). The BET surface area increased approximately 3-fold (to 90 m^2^/g), and the pore volume increased from 0.04 cm^3^/g for BG to 0.11 cm^3^/g for MIL-100­(Fe)@BG. For the intermediate materials, a systematic increase in textural parameters was also observed. After a single deposition cycle, the surface area increased to 62 m^2^/g with a pore volume of 0.08 cm^3^/g, and after three cycles to 74 m^2^/g with a pore volume of 0.10 cm^3^/g (Figure S5). These trends are consistent with the progressive growth of the porous MIL-100­(Fe) layer during the LBL process.

Further characterization by TG analysis revealed a similar thermal decomposition profile for the hybrid material and pristine MIL-100­(Fe) (Figure S6). Therefore, in addition to the weight loss characteristic of BG, the loss between 50 and 530 °C can be attributed to the removal of solvent molecules from the pores, water coordinated to iron trimers and, finally, to the decomposition of the BTC linkers.^53^ This process is accompanied by two exothermic peaks observed at around 321 and 402 °C (Figure S7), which are consistent with the DTA curves of pristine MIL-100­(Fe). A further slight weight loss accompanied by an exothermic peak at 886 °C is likely due to partial crystallization of the glass powders.^54^ Given that the weight loss of pristine BG up to 900 °C was 5%, the weight loss associated with MIL-100­(Fe) degradation in the resulting hybrid is estimated at approximately 14.4% (Figure S6).

As confirmed by HRTEM imaging, MIL-100­(Fe) forms a colloidal shell around the spherical BG nanoparticles (Figure 1e,f) with an average shell thickness of 6.1 ± 0.9 nm (inset in Figure 1f), in line with the nanoscale coating inferred from PXRD data. EDS elemental mapping further indicated the core–shell nature of the hybrid material. Indeed, while Ca and Si signals were mainly detected in the nanoparticle core, Fe was preferentially localized on the nanoparticle surface (Figure 1h). STEM imaging (Figure S8) showed partial surface coverage after one deposition cycle and a denser coating after three cycles, whereas HR-TEM of the sample after five deposition cycles revealed a well-defined MIL-100­(Fe) layer. Altogether, these observations indicate that multiple deposition cycles are required to achieve a uniform coating on the BG nanoparticles.

The progressive formation of the MIL-100­(Fe) layer was further reflected by the surface charge of the particles. In DPBS, the ζ-potential changed from −26.5 ± 1.7 mV for bare BG (−12.1 ± 0.8 mV in water) to −18.2 ± 1.1 mV (−11.7 ± 0.3 mV in water) after one deposition cycle and to −13.0 ± 1.0 mV (−7.1 ± 0.6 mV in water) after three cycles, finally reaching −11.1 ± 1.1 mV (−2.8 ± 0.6 mV in water) for the five-cycle MIL-100­(Fe)@BG material. The parallel measurements in water, a medium of low ionic strength, were used to complement the DPBS data and avoid the strong electrostatic screening that suppresses ζ-potential magnitude in physiological buffers. This continuous shift toward less negative values is consistent with progressive surface coverage by the MIL-100­(Fe) coating. Importantly, the ζ-potential remained negative in all cases, a property widely considered favorable for bone cell attachment and proliferation.^55^

### 3.2 Materials Characterization by X-ray Absorption Spectroscopy

XAS enables comprehensive characterization of selected 3d metals (Ca, Fe) at their L-edges and nonmetals such as O and Si at their K-edges. Through XAS, the spin state and electronic configuration of Ca, Fe, O, and Si can be determined and correlated with other material properties, including local site symmetry, charge state, and orbital occupancy. Therefore, both the hybrid material and its individual components were studied, including the Fe­(III) cluster used in the synthesis of MIL-100­(Fe)@BG.

The XAS spectra show Fe L-edge and O K-edge structures for the [Fe_3_O­(OOCCH_3_)_6_OH]·2H_2_O cluster and MIL-100­(Fe), Ca L-edge and O K-edge structures for BG, and a combination of all these near-edge absorption structures for MIL-100­(Fe)@BG (Figure 1g, S9–S12). These results are in agreement with the elemental analysis determined by the other experimental technique. The normalized Fe L-edge X-ray absorption spectra of Fe­(III) cluster, MIL-100­(Fe) and MIL-100­(Fe)@BG are presented in Figure 1g. The Fe L-edge, corresponds to the electric dipole-allowed 2p→3d transitions, where the 2p-orbital is localized on the Fe. This means that the Fe L-edge intensity is directly proportional to the Fe d-character in the valence orbitals of the metal. Thus, the percentage of metal character in the d-orbitals can be probed by the integrated L-edge intensity.^56–60^ The Fe L-edge spectrum involved the Fe 2p_3/2_→3d and 2p_1/2_→3d transitions as a consequence of the 2p^5^ configuration of excited states (^2^P) and the interaction between an electron’s spin and its orbital angular momentum in the electric field generated by the nucleus. The Fe L-edge XAS spectra consist of two primary sets of peaks corresponding to the L_3_-edge (∼710 eV) and the L_2_-edge (∼720 eV), respectively; the second one is shifted to low-energy peaks by c.a. 10 eV (3/2 × the 2p core spin–orbit coupling) with an intensity ratio of ∼ 2:1, where J = 3/2 and 1/2. The lack of pre-edge peaks at c.a. 707 and 717 eV for low- and high-energy transitions (Figure 1g), respectively, observed mainly for iron­(III) oxalates, catecholates, and hydroxamates^56^ or some low-spin iron­(III) complexes,^57^ may conclude that the [Fe_3_O­(OOCCH_3_)_6_OH]·2H_2_O cluster, MIL-100­(Fe), and the hybrid material with BG have high-spin configuration with lack of π-donating effects of carboxylates that produced weak ligand field. In contrast to the spectra with the pre-edge peaks that characterized Fe­(III) high-spin configuration with π-donating ligands^56^ or Fe­(III) low-spin configuration with back-bonding effects of ligands,^57^ the coordinated carboxylates in Fe­(III) cluster, MIL-100­(Fe), and MIL-100­(Fe)@BG appear to delocalize π electron density mostly over the O–C–O groups, which are weakly involved in π-donor interaction with metal centers.

In the systems with no back-bonding, a lower percentage of metal d-character indicates a system that is more covalent. The shape of the spectrum gives insight not only into the ligand field, but also into the modulation of the multiplet splitting, which is similar to the effects described by the Tanabe-Sugano diagrams for d^N^ metal complexes. Moreover, for the 2p^5^3d^N+1^, final states may also include p-d electron repulsion and spin–orbit coupling. Both peaks of low- (J = 3/2) and high-energy (J = 1/2) are split into doublets and are separated by c.a. 1.3, 1.5, 1.8 eV for hybrid, Fe­(III) cluster and MIL-100­(Fe), respectively (Figure 1g). These energy gaps are only slightly higher than 1.2 eV, responsible for p-d electron repulsion between the 2p core unpaired electron and the 3d^N‑1^ unpaired electrons in the excited state (^6^P) in the case of a weak O_h_ ligand field. The energy separation is a consequence of mixing the lowest energy term ^6^F with the ^6^P excited term, where only the transition from ^6^S ground state is electric dipole allowed, and is a function of p core electron-d electron interactions. At low 10 Dq, there is little intensity of the ^6^F state, and the two states are separated by p-d repulsion (1.2 eV). However, the difference in energy between the two peaks of low-energy doublets for XAS spectra presented in Figure 1g (approximately 1.2 eV) can be attributed to the p-d repulsion, suggesting that the splitting of these two states becomes slightly influenced by the ligand field strength (10 Dq). Nevertheless, this additional ligand-field effect is not strong, which is consistent with the lack of pre-edge peak in Fe L-edge XAS spectra.

On the other hand, the O K-edge spectra of all materials show a dominant peak at 532 eV that can be assigned to the π* resonance, followed by the σ* resonance at a higher energy of around 540 eV (Figure S10). In the spectra of MIL-100­(Fe), a peak at 534.5 eV, with an energy difference from the main peak of 2.5 eV, has been reported and assigned to the 1s → π* excitation of OH oxygen in the carboxylate group, observed mostly on the surface of the material.^61^ In addition, MIL-100­(Fe)@BG and BG show a similar feature around 534–535 eV. A probable contribution from residual water is expected in the energy range 537–540 eV, and in the spectra, these transitions are observed as broad signals.^62^

The Ca L-edge XAS spectra did not change significantly, and no energy shifts were observed when comparing MIL-100­(Fe)@BG with pristine BG (Figure S11), suggesting that calcium ions did not influence the bonding with MIL-100­(Fe). The intensity of satellite prepeaks and main 2p-3d transition peaks (∼349 eV and ∼352 eV) remained constant for both materials. The energy gap between each prepeak and the corresponding main peak is c.a. 1 eV, suggesting that calcium exists in octahedral O_h_ local symmetry in both materials. The second order diffraction from the Fe absorption edge is responsible for the appearance of less intense peaks at 354 and 361 eV in Figure S11b. These peaks are identified at energies two times lower than the energy of the initial L-edge XAS Fe peaks.

### 3.3 Bioactivity Assay

The bioactivity of MIL-100­(Fe)@BG was assessed in two widely used physiological media with distinct ionic compositions and buffering characteristics: calcium-free DPBS and SBF. DPBS contains ∼ 10 mM phosphate and provides strong buffering, favoring rapid supersaturation toward calcium phosphate. In contrast, SBF contains Mg^2+^ and HCO_3_^–^, which compete with Ca^2+^ and inhibit HA crystallization, while its tighter buffering capacity suppresses supersaturation.^30,63^ For this reason, comparing the hybrid material in both media provides complementary insight into its biomineralization behavior. Calcium-free DPBS was specifically selected to determine whether Ca released solely from the hybrid system (via dissolution of the BG core) is sufficient to trigger HA formation. In contrast, SBF contains Ca^2+^ ions and is widely used to evaluate bioactivity under conditions that mimic ion concentrations in human plasma. In both media, MIL-100­(Fe)@BG was tested in parallel with pristine BG to determine how the MIL-100­(Fe) coating influences HA growth kinetics.

PXRD pattern of MIL-100@BG incubated in DPBS for 4 h revealed two broad reflections at ca. 26° and 32° 2θ, corresponding to the (002) and (211/112) planes of HA, indicating the early stage of crystallization (Figure 2a). After 72 h, additional reflections appeared at ca. 40° (310), 47° (222), 50° (213), and 53° (004), confirming progressive HA growth (Figure 2a). In comparison, these reflections emerged earlier in the pristine BG after just 4 h of incubation (Figure 2b). FTIR spectra corroborated these findings: the O–P–O bending bands at 604 cm^–1^ and 563 cm^–1^ characteristic of HA developed within 4 h for both materials (Figure 2c,d). Also noteworthy is the change in the shape and position of the most intense Si–O–Si stretching band, which shifts from its original maximum at 1098 cm^–1^ to a broader envelope with a shoulder at 1034 cm^–1^ after 4 h, characteristic of P–O stretching vibrations in HA. Notably, after 4 h incubation of the hybrid material, no FTIR bands or PXRD peaks from the MIL-100­(Fe) component were detected (Figure 2a,c), indicating the rapid degradation of the MOF under these conditions. However, ICP-OES analysis showed that the Fe content in the recovered solids remained constant throughout the incubation period (Figure 2e), and Fe release into solution, measured up to 72 h, remained below detection limits. This confirms that Fe^3+^ ions remain incorporated in the forming mineral phase rather than being leached out. In contrast, the P content increased sharply to 6–7 wt % after only 4 h, consistent with rapid HA nucleation, and later reached ∼11 wt % for MIL-100­(Fe)@BG (maximum at 7d) and ∼9 wt % for BG, with the plateau reached by 24 h and maintained up to 21 days. Simultaneously, the Si content decreased from ∼24 wt % to ∼5 wt % in MIL-100­(Fe)@BG and to ∼9 wt % in BG, reflecting the dissolution of the silicate network (Figure 2e,f). These transformations were accompanied by rising pH, increasing from 7.40 to 7.97 after 4 h and to 8.33 after 72 h for MIL-100­(Fe)@BG (and more strongly for BG, reaching 9.99 at 72 h), consistent with ion exchange and silicate dissolution (Figure S13a). In parallel, ζ-potential measurements of MIL-100­(Fe)@BG during incubation in DPBS revealed rapid interfacial evolution: the potential shifted from −11.1 mV at the start of incubation to −12.7 mV at 30 min and further −15.5 mV at 4 h, after which it remained nearly constant up to 24 h (Figure S14, Table S2). This behavior reflects early stage MOF decomposition and the onset of surface mineralization.

**Figure 2:**
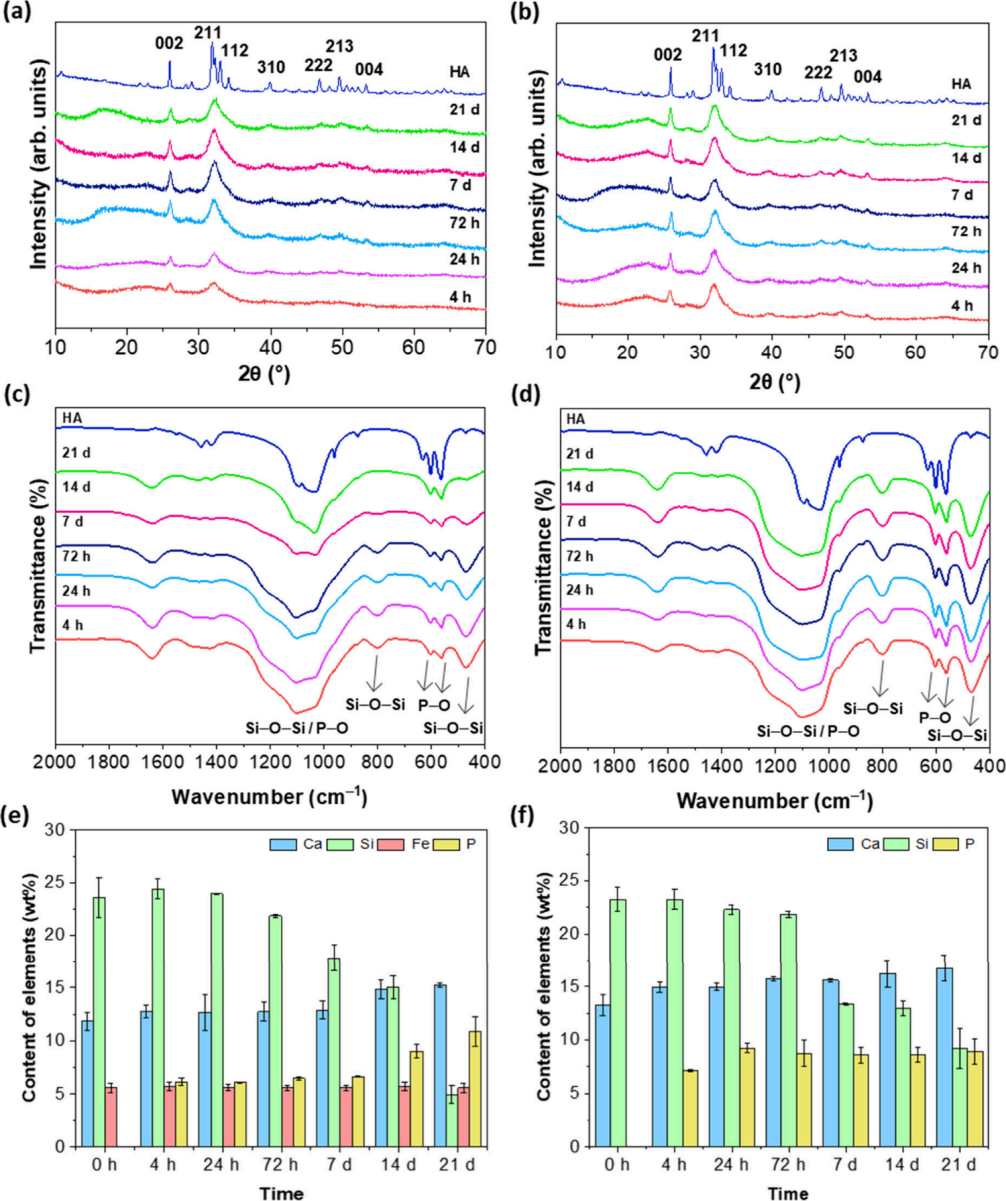
PXRD patterns (a,b), FTIR spectra (c,d) and compositional analysis monitored by ICP-OES (e,f) for MIL-100­(Fe)@BG (a,c,e), and BG (b,d,f) exposed to the bioactivity test (DPBS, pH 7.4, 37 °C, 4 h - 21 days).

In contrast to DPBS, HA formation in SBF proceeded more slowly. PXRD, FTIR and ICP-OES all indicated delayed mineralization: the first weak indications of HA appeared after 24 h of incubation, becoming more pronounced at longer time points (Figures S15–S17). Quantitatively, the P content increased only modestly, from ∼1 wt % after 4 h to ∼9–10 wt % after 21 d (Figure S15), and the dissolution of the silicate network, as well as the evolution of Ca content, followed different trends for BG and MIL-100­(Fe)@BG. These slower kinetics arise from the distinct ionic composition mentioned above, which inhibits HA crystallization and stabilizes amorphous precursors. Additionally, CaCO_3_ was detected as a competing mineralization pathway (Figure S16b), further delaying HA formation. Consistent with these observations, pH changes in SBF were more moderate, rising only to 7.89 (4 h) and 8.02 (72 h) for MIL-100­(Fe)@BG and to 7.73 (4 h) and 7.84 (72 h) for BG (Figure S13b). As in DPBS, the Fe content of MIL-100­(Fe)@BG remained constant throughout the experiment (Figure S15a), indicating the absence of Fe leaching despite the rapid chemical decomposition of MIL-100­(Fe) crystallinity observed by PXRD and FTIR. XPS analysis further verified that Fe remained in the +3 oxidation state after 7 d of incubation in both media (Figures S18–S19, Table S3), and that the observed P 2p binding energies (133.2–133.3 eV) were consistent with HA formation.^64^

### 3.4 Monitoring of Hybrid Conversion by HRTEM

To investigate in detail the structural and morphological changes occurring in MIL-100­(Fe)@BG under simulated physiological conditions, HRTEM and STEM-EDS analyses were performed after the initial (after 4 h) and final (after 21 d) stages of the bioactivity test. After only 4 h of incubation, HRTEM images and FFT analysis revealed the formation of needle-like HA nanocrystallites on the hybrid surface (Figure 3a-h). Periodic fringes corresponding to several HA lattice planes were identified (Figure 3e,g), and STEM-EDS mapping confirmed the colocalization of Ca and P, with their abundance increasing over time (Figure 3i,j). Similar behavior was observed for MIL-100­(Fe)@BG incubated in SBF and for pristine BG in both media (Figures S20–S25).

**Figure 3:**
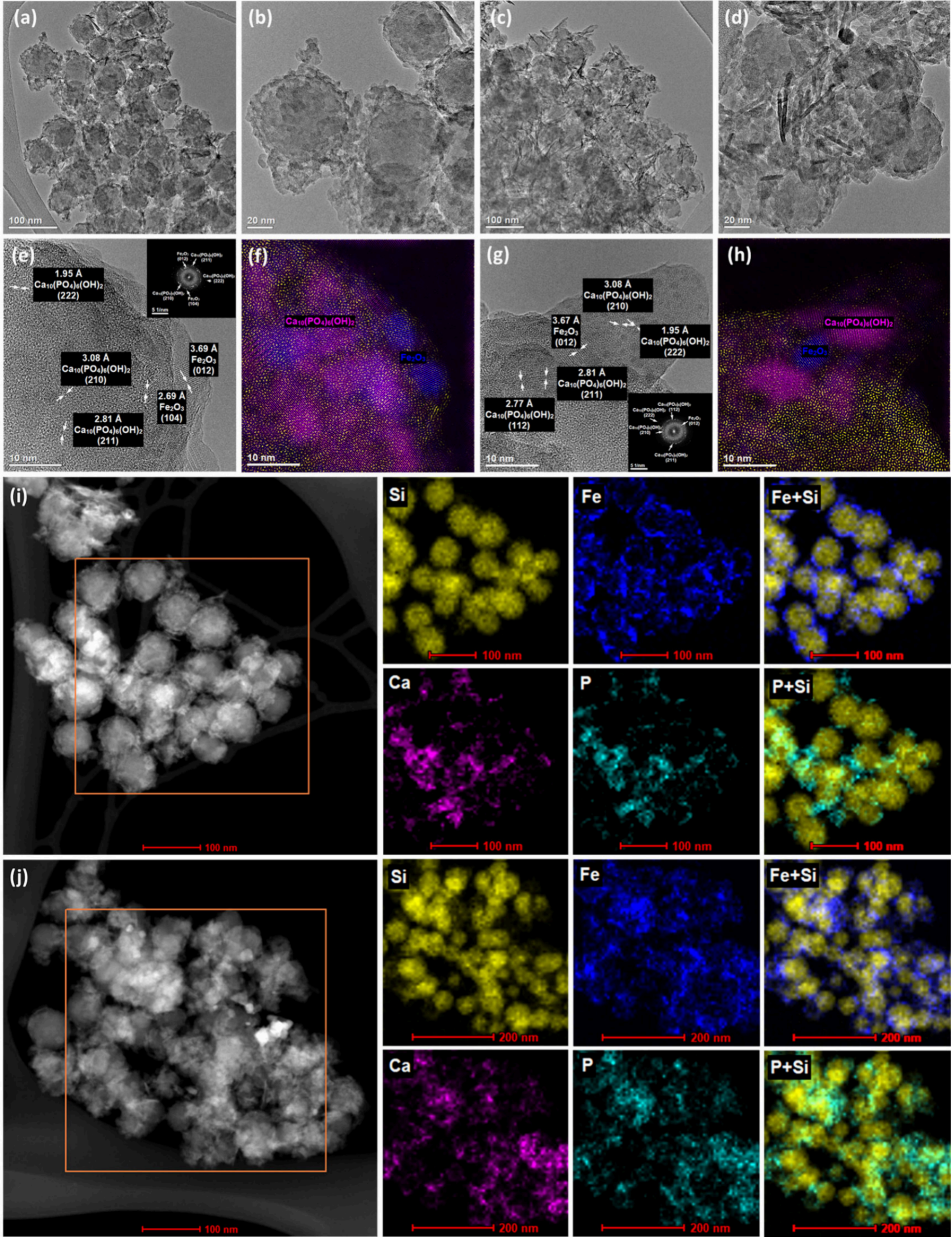
HRTEM images (a-d) with phase analysis and FFT (e-h), and EDS elemental mapping (i,j) of MIL-100­(Fe)@BG after 4 h (a,b,e,f,i) and 21 d (c,d,g,h,j) of immersion in DPBS (pH 7.4, 37 °C).

In parallel, a pronounced reduction in particle size (exceeding a 2-fold decrease) was observed, particularly for MIL-100­(Fe)@BG in SBF (Figures S24) and for BG in DPBS (Figure S20), accompanied by a loss of spherical morphology. This behavior is fully consistent with the well-established degradation mechanism of BGs, which undergo ion-exchange-driven dissolution and progressive erosion of the silicate network under physiological conditions. Such degradation-induced size reduction may influence biological performance, including circulation, clearance and biodistribution, especially because smaller particles are known to exhibit longer circulation times and enhance tissue penetration. A dedicated biological and pharmacokinetic assessment will be the focus of future studies.

Beyond these morphological changes, important insights were obtained regarding the evolution of the MIL-100­(Fe) component. Although no MIL-100­(Fe) reflections or FTIR bands remained detectable after 4 h, ICP-OES analyses showed that Fe content in the solid phase did not decrease (Figure 2e, Figure S15a). At this early stage, the core–shell architecture was still discernible in STEM-EDS maps (Figure 3i). However, HRTEM/FFT analysis revealed nanoscale crystalline nanoparticles within the former shell, exhibiting interplanar spacings of 2.69 Å and 3.69 Å, corresponding to the (104) and (012) planes of α-Fe_2_O_3_ (Figure 3e,f). The resulting Fe_2_O_3_ nanoparticles (2–6 nm) are therefore the solid-state transformation products of MIL-100­(Fe) formed under physiological conditions. Amorphous Fe-phosphate species, commonly reported for MIL-100­(Fe) degradation in phosphate-containing buffers, cannot be excluded; however, crystalline Fe_2_O_3_ is clearly identified in the samples. Prolonged incubation (21 d) led to the further disintegration of the initial shell (Figure 3j). EDS maps indicated that Fe remained colocalized with Ca and P, suggesting that Fe_2_O_3_ nanoparticles become anchored to newly formed HA crystallites (Figure S26). A similar transformation of MIL-100­(Fe) into Fe_2_O_3_ was observed for MIL-100­(Fe)@BG incubated in SBF (Figure S24).

To contextualize these findings, the stability of pristine MIL-100­(Fe) was examined under identical incubation conditions (1.5 mg/mL, DPBS, 37 °C, 100 rpm). Consistent with previous reports showing phosphate-induced linker exchange and framework breakdown,^65–67^ the crystalline structure of MIL-100­(Fe) deteriorated rapidly: PXRD reflections diminished significantly within 4 h, accompanied by the appearance of an FTIR band at ∼1035 cm^–1^ characteristic of Fe-phosphate species (Figure S27a,b). Although weak MIL-100­(Fe) reflections were still detectable after 72 h, they disappeared completely after 7 days. Despite this loss of long-range order, Fe release remained minimal (a maximum of 1.27 ppm after 24 h, ∼0.54% of total Fe content), confirming that degradation proceeds via a solid-state transformation rather than dissolution (Figure S27c). Significantly, the pH of the incubation medium decreased from 7.4 to 5.8 within the first 7 days, consistent with the release of acidic carboxylate fragments during linker hydrolysis (Figure S27d). This acidification further promotes framework destabilization and aligns with the degradation behavior expected for MIL-100­(Fe) in phosphate-rich environments. At longer incubation times (21 d), HRTEM/FFT analysis of the recovered solids revealed nanoscale particles exhibiting interplanar spacings of 2.19 Å and 2.69 Å, matching the (113) and (104) planes of α-Fe_2_O_3_ (Figure S28). Weak FTIR bands at 549 and 475 cm^–1^, consistent with Fe–O vibrations, further supported this assignment (Figure S27a). These independent stability experiments validate the behavior observed in the hybrid system and substantiate the identification of α-Fe_2_O_3_ nanoparticles as transformation products of MIL-100­(Fe) under phosphate-rich physiological conditions.

Finally, these results allow us to propose a plausible mechanism for the MIL-100­(Fe) → Fe_2_O_3_ transformation in the MIL-100­(Fe)@BG hybrid material. MIL-100­(Fe) is constructed from trimeric μ_3_-oxo Fe­(III) clusters bridged by 1,3,5-benzenetricarboxylate linkers. Upon immersion in physiological media such as DPBS or SBF, the framework undergoes hydrolysis and phosphate-driven ligand exchange, progressively cleaving Fe–carboxylate bonds and releasing hydrated Fe–OH species from the cluster environment. Although ICP-OES confirms that Fe does not dissolve into the solution, these Fe-hydroxo species can condense on the BG surface, which becomes enriched in silanol groups as a result of ion-exchange-driven dissolution of the BG. This heterogeneous nucleation pathway favors the formation of poorly ordered Fe-oxyhydroxide intermediates (ferrihydrite/FeOOH-like), which subsequently dehydrate into α-Fe_2_O_3_ at 37 °C, consistent with widely reported FeOOH → Fe_2_O_3_ transformation routes in aqueous media.^68^

### 3.5 The Influence of the MIL-100­(Fe) Layer on Biocompatibility and Osteointegration of BG Nanoparticles

The *in vitro* cytocompatibility of BG nanoparticles and the MIL-100@BG hybrid was evaluated using HDF and MC3T3 cells as model normal cell lines. After 24 h of incubation, BG nanoparticles in the tested concentration range (1–1000 μg/mL) did not induce significant changes in HDF viability (Figure 4a). Extending the incubation time to 72 h led to a decrease in viability at the highest concentration tested (1000 μg/mL), from 88 to 65% (Figure S29a). Coating BG nanoparticles with MIL-100­(Fe) did not impair short-term cytocompatibility. After 24 h, the hybrid remained well tolerated up to 500 μg/mL, with viability decreasing to 63% only at 1000 μg/mL (Figure 4a). Notably, prolonged exposure (72 h) markedly improved the biocompatibility profile of MIL-100­(Fe)@BG: viability increased to nearly 100% even at the highest tested concentration (Figure S29a). Additional long-term assays conducted up to 21 days corroborated this trend, again showing reduced HDF survival only at 1000 μg/mL, consistent with the behavior observed at earlier time points (Figure 4b).

**Figure 4:**
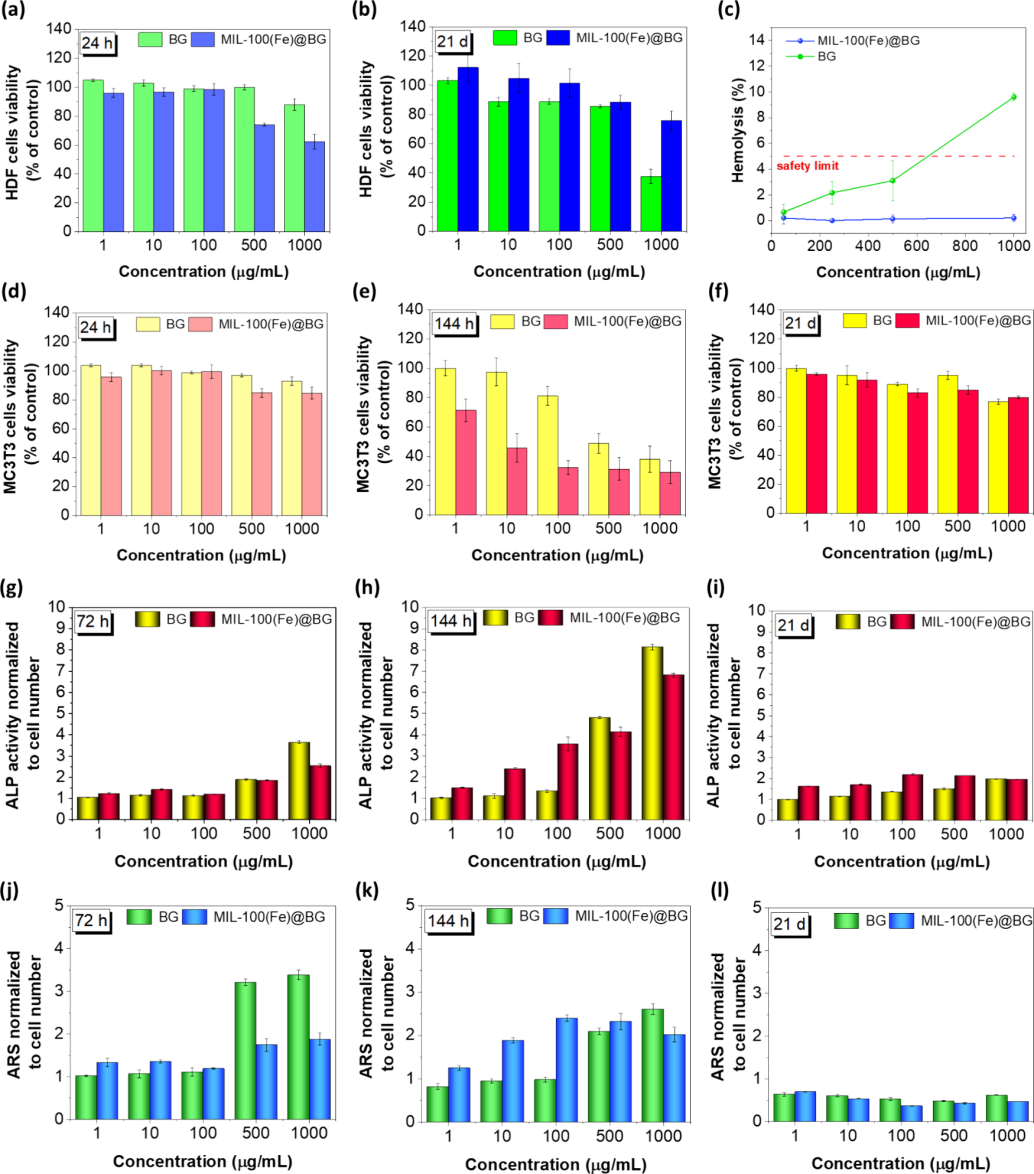
Determination of the biomedical profile of BG and MIL-100­(Fe)@BG by evaluation of: viability of human dermal fibroblasts (HDF) cells (a,b); hemolysis study (c); viability of mouse osteoblast precursors (MC3T3) (d,e,f); osteointegration markers, including alkaline phosphatase (ALP) activity (g,h,i) and alizarin red staining (ARS) (j,k,l) in mouse osteoblast precursor cells (MC3T3) cultured in the presence of tested materials.

In contrast, MC3T3 cells exhibited high tolerance to both BG and MIL-100­(Fe)@BG during the short-time exposure, with viability remaining above 85% after 24 h at all concentrations (Figure 4d) and ranging from ∼80% for BG to ∼70% for MIL-100­(Fe)@BG after 72 h at the highest concentration tested (Figure S29b). One possible explanation for the different responses of HDF and MC3T3 cells at 1000 μg/mL is the difference in their proliferation rates: HDF cells proliferate faster and more intensively, making them more sensitive to growth limitations. At extended time points (144 h), a decrease in MTT reduction was observed, particularly in cultures treated with the hybrid material (Figure 4e). This decrease is attributed to reduced metabolic activity and proliferation rate associated with early osteogenic differentiation rather than cytotoxicity, as supported by concomitant increases in ALP activity and extracellular matrix mineralization. Indeed, both BG and MIL-100­(Fe)@BG promoted osteogenic commitment of MC3T3 cells, with the hybrid inducing a stronger response, as evidenced by elevated ALP activity and more intense ARS observed after 72 and 144 h of exposure (Figure 4g,h,j,k). However, under long-term culture, the cells remain in static and relatively “artificial” conditions, with continuous exposure to the materials, which may be not sufficient to sustain the ongoing osteogenesis. This likely explains why the osteogenic commitment appears diminished after 21 days (Figure 4 i,l). Importantly, this does not imply that the cells have lost their ability to differentiate; rather, under *in vivo* conditions they would be exposed to a broader spectrum of osteogenic cues derived, among others, from surrounding bone cells. Consistent with this interpretation, the extended 21-day assays showed that MC3T3 cells maintained high viability, remaining above 80% even at the highest concentration (1000 μg/mL) (Figure 4f).

Hemocompatibility testing further demonstrated the beneficial effect of MIL-100­(Fe) coating. BG nanoparticles exhibited low hemolysis at 250 μg/mL (2.2%) and 500 μg/mL (3.1%), consistent with the best reported values for sol–gel BGs (Figure 4c). At 1000 μg/mL, BG approached the threshold of marked hemolysis (9.6%). By contrast, MIL-100­(Fe)@BG induced only 0.2% hemolysis at this concentration, i.e., well within the range considered nonhemolytic (<5%),^69^ indicating that the presence of the MOF layer significantly enhances the blood compatibility of the system. To our knowledge, this is the first demonstration that a MOF/BG interface can reduce hemolytic activity.

Biocompatibility data reported to date for MOF@BG systems have largely focused on scaffolds or disk-shaped materials rather than powders.^27–29^ For example, Fe-MOF-functionalized MBG scaffolds supported adhesion of human bone marrow-derived mesenchymal stem cells (hBMSCs),^27^ ZIF-8@VAN@BG scaffolds promoted rat bone marrow mesenchymal stem cells (rBMSC) proliferation and osteogenic differentiation,^28^ and Ag@Cu-MOF@BG disks were nontoxic in indirect tests against HDFs.^29^ The behavior of MIL-100­(Fe)@BG reported here is fully consistent with these findings. Overall, the integration of MIL-100­(Fe) with BG does not compromise the intrinsic cytocompatibility of BG, and enhances its hemocompatibility, while maintaining specific biological capability for inducing osteogenesis, thereby supporting the potential of MIL-100­(Fe)@BG for bone-regenerative applications.

### 3.6 Antibacterial Activity

Bone infection (osteomyelitis) remains a serious clinical challenge following complex fractures or orthopedic implant procedures.^70^ Because *S. aureus* and *E. coli* are among the most common causative pathogens, these strains were selected to assess the antibacterial activity of BG and MIL-100­(Fe)@BG. The results demonstrated that pristine BG nanoparticles exhibited no detectable antibacterial activity. In contrast, coating BG with MIL-100­(Fe) significantly reduced bacterial viability, decreasing survival to 73% for *S. aureus* and 58% for *E. coli* (Figure 5a,b). The stronger antibacterial effect against *E.coli* might be a consequence of its higher susceptibility to oxidative and pH-mediated stress compared to *S. aureus.* This observation aligns well with previous studies showing that ROS-driven antibacterial outcomes are species-dependent rather than strictly determined by Gram classification.^71,72^ To contextualize these values, previous MOF@BG systems incorporating exogenous antibacterial agents show considerably stronger activity. For example, Ag@Cu-MOF@BG composite produced inhibition zones of 8–10 mm and ≥ 3-log bacterial reduction due to Ag^+^ release,^29^ while ZIF-8@VAN@BG scaffolds achieved 62.5–93.5% inhibition depending on vancomycin loading.^28^ Importantly, these systems rely on silver or antibiotic release, whereas the antibacterial performance of MIL-100­(Fe)@BG is achieved without external drugs.

**Figure 5:**
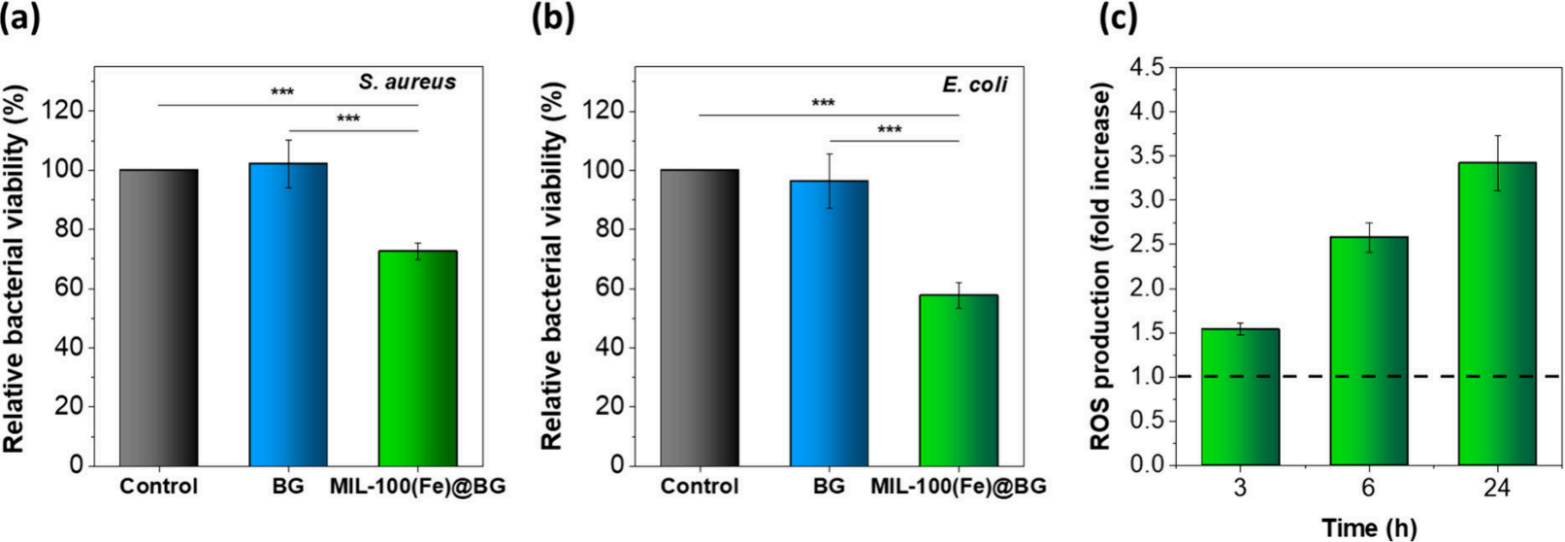
Antibacterial activity of BG and MIL-100­(Fe)@BG against *S. aureus* (Gram-positive) (a) and *E. coli* (Gram-negative) after 24 h of incubation. When analyzed by Bonferroni’s test, the asterisks denote a significant difference (***p < 0.001). (c) Effect of MIL-100­(Fe)@BG on the levels of reactive oxygen species (ROS) in *E. coli* after 3, 6, and 24 h of incubation. The results are expressed as fold change relative to control (bacteria + DCFH-DA), which was set to 1.0. The horizontal dashed line represents ROS production in the control group. Fluorescence values were corrected by subtracting the PBS blank and the autofluorescence of bacteria without the probe.

To date, MIL-100­(Fe) has been used as a component of diverse antibacterial composites, primarily for food-packaging applications, such as Carvacrol@MOF,^73^ MIL-100­(Fe)-TTO,^74^ where TTO: tea tree essential oil, and MIL-100­(Fe)/chitosan-embedded polyvinylidene fluoride.^75^ In MIL-100­(Fe)@X systems (X = amoxicillin and potassium clavulanate,^76^ 3-azido-d-alanine^77^), antimicrobial effects originate from the payload rather than the MOF. AgI/MIL-100­(Fe) heterojunctions exhibit photocatalytic ROS generation and efficiently inactivate *E. coli* and *S. aureus*.^78^ However, MIL-100­(Fe) alone displays limited antibacterial activity, largely due to its high structural stability in short-term aqueous exposure and minimal ion release. In the hybrid system, the situation differs fundamentally. The colloidal MIL-100­(Fe) shell, markedly less stable than hydrothermally synthesized MIL-100­(Fe), undergoes rapid partial structural degradation under physiological pH, forming α-Fe_2_O_3_ nanoparticles. Importantly, although Fe_2_O_3_ has previously been incorporated into BG for other biomedical applications (such as enhanced apatite formation, ROS-mediated therapeutic effects, and magnetic hyperthermia),^79–82^ to the best of our knowledge, no studies have reported antibacterial properties of Fe_2_O_3_-containing BG systems. In parallel, dissolution of the BG core releases Ca^2+^ and silicate ions, producing local alkaline shifts known to destabilize bacterial envelopes.^19^ The synergy of these processes creates conditions conducive to antibacterial activity that are unique to the MIL-100­(Fe)@BG hybrid system.

To clarify the contribution of oxidative stress, intracellular ROS levels in *E. coli* were quantified. ROS production steadily increased, reaching 1.54-fold after 3 h, 2.58-fold after 6 h, and 3.42-fold after 24 h (Figure 5c). This strong time-dependent enhancement correlates with bacterial inactivation. Iron-containing materials, particularly α-Fe_2_O_3_ nanoparticles, are widely used as catalysts for Fenton-like reactions.^83,84^ In biological environments, H_2_O_2_ naturally produced by bacteria under stress can be activated to generate ROS, with hydroxyl radicals (·OH) considered to play a major role in oxidative damage to bacterial membranes, proteins, and genomic material.^71^

Taken together, the antibacterial behavior of MIL-100­(Fe)@BG is governed by a drug-free, transformation-driven mechanism most likely involving the following steps: (i) structural evolution of the MIL-100­(Fe) shell into catalytically active α-Fe_2_O_3_ nanoparticles; (ii) ion release from the BG matrix; and (iii) the resulting oxidative and pH-mediated stress, which is known to impair bacterial envelope function and viability. Additional studies will be necessary to refine our understanding of the relative contribution of each process.

## 4 Conclusions

In this work, we demonstrated the synthesis of a novel core–shell hybrid material, MIL-100­(Fe)@BG, obtained through a LBL deposition of a colloidal MIL-100­(Fe) shell on BG nanoparticles. Comprehensive physicochemical characterization confirmed the successful formation of a homogeneous nanoscale coating and revealed its rapid transformation under simulated physiological conditions. Bioactivity assays performed in calcium-free DPBS and SBF showed that MIL-100­(Fe)@BG readily induces the formation of nanocrystalline HA, with kinetics that reflect the distinct ionic composition of the two media. HRTEM analyses further showed the structural evolution of the hybrid, including progressive BG dissolution and conversion of the MIL-100­(Fe) shell into nanometric α-Fe_2_O_3_ anchored to the forming HA. These insights clarify the fate of Fe-MOF coatings under physiological conditions and highlight the different degradation pathways of MIL-100­(Fe) when immobilized on a reactive biomaterial surface.

*In vitro* studies confirmed that MIL-100­(Fe)@BG is biocompatible with both fibroblasts and osteoblast-like cells, supporting cell viability and promoting osteogenic differentiation at early time points. Importantly, the hybrid exhibited excellent hemocompatibility, causing <0.2% hemolysis even at 1000 μg/mL. Beyond its bioactivity and cytocompatibility, the material exhibited intrinsic antibacterial activity against *S. aureus* and *E. coli* without the need for external therapeutic agents. Mechanistic analysis suggests that antibacterial performance arises from the transformation-driven generation of α-Fe_2_O_3_ nanoparticles, coupled with BG-derived ion release, which collectively induce oxidative stress and impair bacterial viability.

Overall, the presented results show that the MOF/biomaterial interface endows BG nanoparticles with additional functionalities, including enhanced bioactivity, biocompatibility, hemocompatibility, and intrinsic antibacterial behavior. These synergistic properties make MIL-100­(Fe)@BG a promising candidate for applications requiring simultaneous bone regeneration and antimicrobial protection, while also providing a foundation for future optimization of MIL-100­(Fe)–based hybrid systems.

## Supplementary Material



## Notes

TEM, HRTEM, and STEM images with particle size analysis; EDS elemental mapping; FTIR spectra; UV–Vis diffuse reflectance spectra and Tauc plots; N_2_ adsorption–desorption isotherms; TG and TG–DTA analyses; XPS spectra with detailed peak fitting and chemical assignments before and after incubation; XAS spectra; PXRD patterns; ζ-potential measurements; pH monitoring; ICP-OES composition analysis during bioactivity testing in SBF; cytocompatibility data for HDF and MC3T3 cells after 72 h of incubation and additional stability studies of MIL-100­(Fe) (PDF)
