# Supplementary material for: Core–Shell Structures of Bioactive Glass Nanoparticles and MIL-100 Framework: Properties and Biomedical Applications
Source: ACS Biomater Sci Eng. 2026 Jan 29;12(2):820–36. doi: 10.1021/acsbiomaterials.5c01261 (PMC13296726; doi:10.1021/acsbiomaterials.5c01261)
Supplement: Supplementary file 1 [file ab5c01261_si_001.pdf]

Supporting Information

Core–shell structures of bioactive glass nanoparticles and MIL-100 framework: properties and biomedical applications

Marzena Fandzloch [a*], Beata Barszcz [a], Andrada-Ioana Damian-Buda [b], Joanna Wiśniewska [c], Katarzyna Roszek [d], Grzegorz Słowik [e], Anna Jaromin [f], Magdalena Zaremba-Czogalla [f], Muhammad Asim Akhtar [b], Aldo R. Boccaccini [b]

[a] *Institute of Low Temperature and Structure Research, Polish Academy of Sciences, Okólna 2, 50-422, Wrocław, Poland*

[b] *Department of Material Science and Engineering, Institute of Biomaterials, University of Erlangen-Nuremberg, 91058 Erlangen, Germany*

[c] *Faculty of Chemistry, Nicolaus Copernicus University in Toruń, Gagarina 7, 87-100 Toruń, Poland*

[d] *Faculty of Biological and Veterinary Sciences, Nicolaus Copernicus University in Toruń, Lwowska 1, 87-100 Toruń, Poland*

[e] *Department of Chemical Technology, Institute of Chemical Sciences, Faculty of Chemistry, Maria Curie-Sklodowska University in Lublin, 3 Maria Curie-Skłodowska Square, 20-031, Lublin, Poland*

[f] *Department of Lipids and Liposomes, Faculty of Biotechnology, University of Wrocław, F. Joliot-Curie 14a, 50-383 Wrocław, Poland*

*corresponding author: m.fandzloch@intibs.pl

Index

Figure S1……………………………………………………………………………………3
Figure S2……………………………………………………………………………………4
Figure S3……………………………………………………………………………………5
Table S1……………………………………………………………………………………6
Figure S4……………………………………………………………………………………7
Figure S5……………………………………………………………………………………8
Figure S6……………………………………………………………………………………9
Figure S7……………………………………………………………………………………10
Figure S8……………………………………………………………………………………11
Figure S9……………………………………………………………………………………12
Figure S10…………………………………………………………………………………13
Figure S11…………………………………………………………………………………14
Figure S12…………………………………………………………………………………15
Figure S13…………………………………………………………………………………16
Figure S14…………………………………………………………………………………17
Table S2……………………………………………………………………………………17
Figure S15…………………………………………………………………………………18
Figure S16…………………………………………………………………………………19
Figure S17…………………………………………………………………………………20
Figure S18…………………………………………………………………………………21
Figure S19…………………………………………………………………………………22
Table S3……………………………………………………………………………………23
Figure S20…………………………………………………………………………………24
Figure S21…………………………………………………………………………………24
Figure S22…………………………………………………………………………………25
Figure S23…………………………………………………………………………………25
Figure S24…………………………………………………………………………………26
Figure S25…………………………………………………………………………………26
Figure S26…………………………………………………………………………………27
Figure S27…………………………………………………………………………………28
Figure S28…………………………………………………………………………………29
Figure S29…………………………………………………………………………………30
References…………………………………………………………………………………31

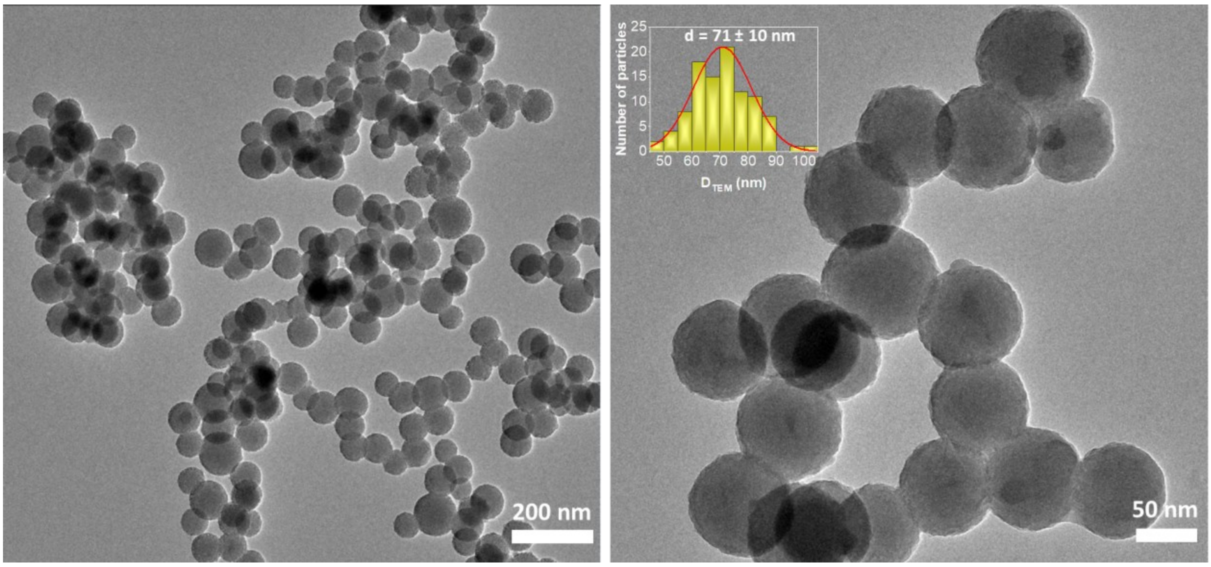

Figure S1. TEM images of BG with the insertion of a size distribution histogram.

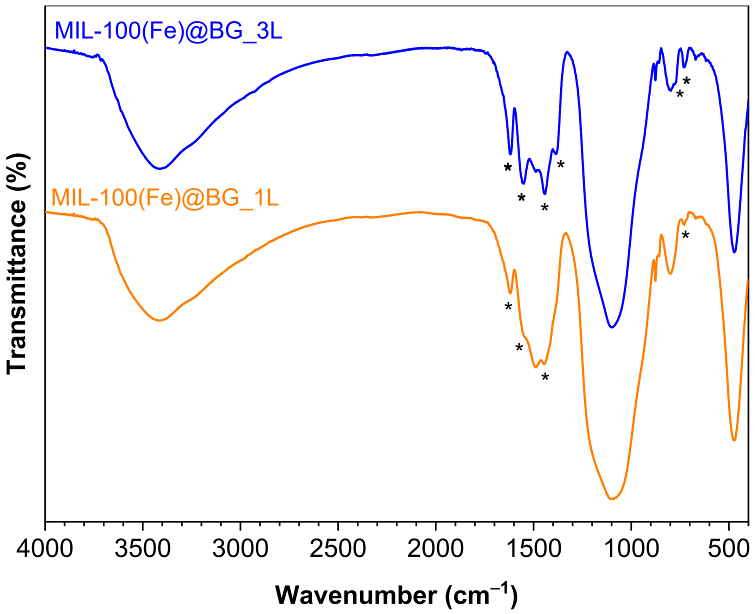

Figure S2. FTIR spectra of MIL-100(Fe)@BG prepared using one and three deposition layers of MIL-100(Fe). Bands attributed to MIL-100(Fe) are marked with asterisks. The band observed predominantly after one deposition cycle at 1491 cm^{-1} can be assigned to the asymmetric stretching of CO_3^{2-} groups originating from BG. For MIL-100(Fe)@BG obtained after five deposition cycles, the reader is referred to Figure 1b in the main manuscript.

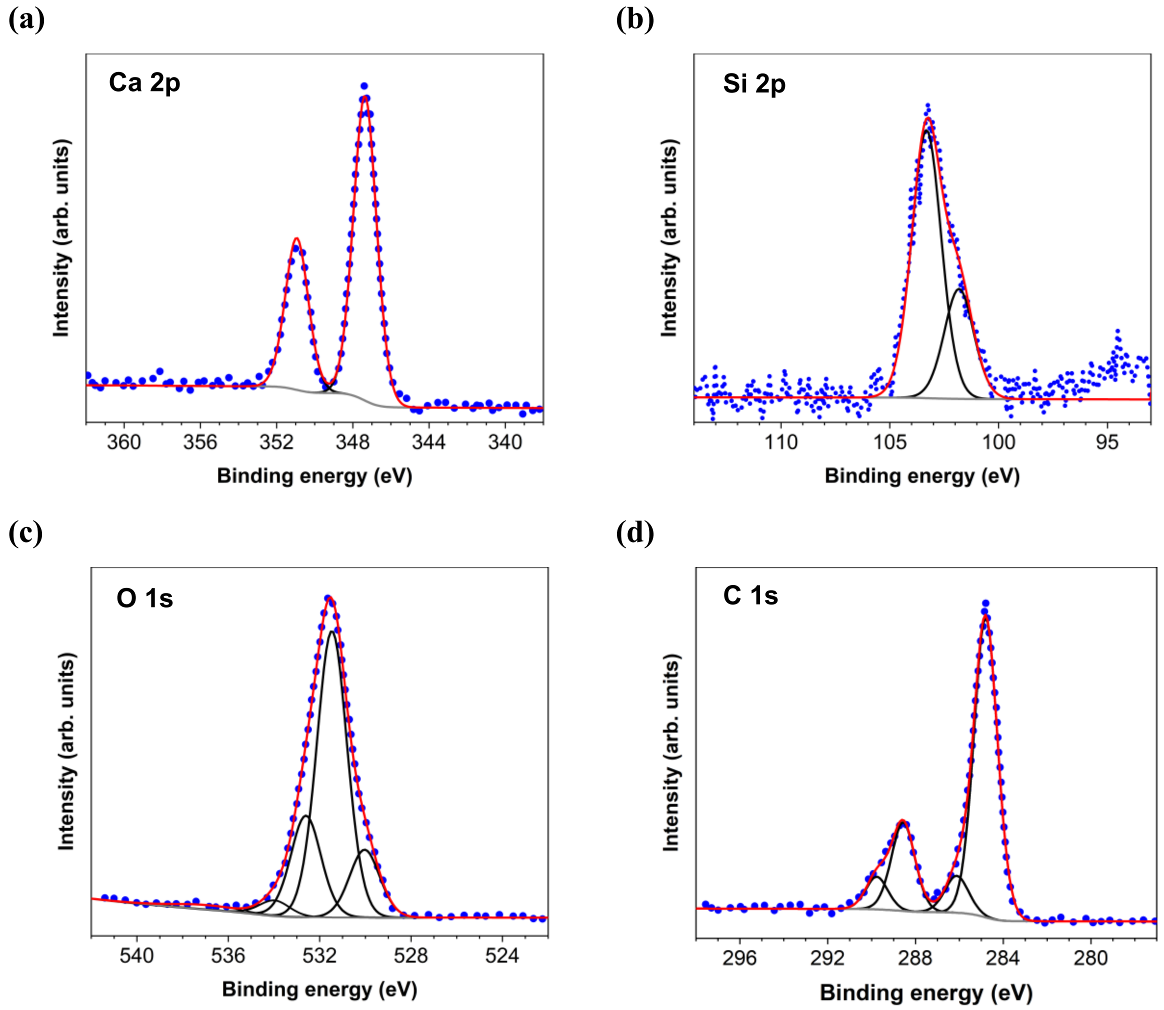

Figure S3. High-resolution XPS spectra of MIL-100(Fe)@BG: Ca 2p (a), Si 2p (b), O 1s (c), and C 1s (d).

Table S1. XPS fitting results showing binding energy values and corresponding chemical assignments for key elements in MIL-100(Fe)@BG.

Peak	Binding energy (eV)	Assignment	Area (%)	Reference
Fe 2p	711.1	Fe(III) $2p_{3/2}$	29.7	[1]
	712.7	Fe(III) $2p_{3/2}$	27.2	
	718.8	Satellite	14.6	
	724.6	Fe(III) $2p_{1/2}$	13.5	
	726.2	Fe(III) $2p_{1/2}$	14.9	
Ca 2p	347.4	Ca(II) $2p_{3/2}$	50.7	[2]
	350.9	Ca(II) $2p_{1/2}$	49.3	
Si 2p	103.3	Si–O–Si	70.9	[3]
	101.8	Si–O–Ca	29.1	[4]
O 1s	530.0	O–Fe	14.4	[1]
	531.5	Si–O–Ca / R–O–C=**O**	60.8	[1], [4-5]
	532.6	C–OH / Si–O–Si	21.5	[1], [4-5]
	534.0	O=C–**O–**R / Si–OH	3.3	[1], [5]
C 1s	284.8	C=C / C–C / C–H	65.7	[1,6]
	286.1	C–O	8.1	[6]
	287.6	C=O	0.1	[7]
	288.6	R–O–C=O	19.4	[1]
	289.8	$\pi \rightarrow \pi^*$/ CO_3^{2-}	6.7	[1], [8]

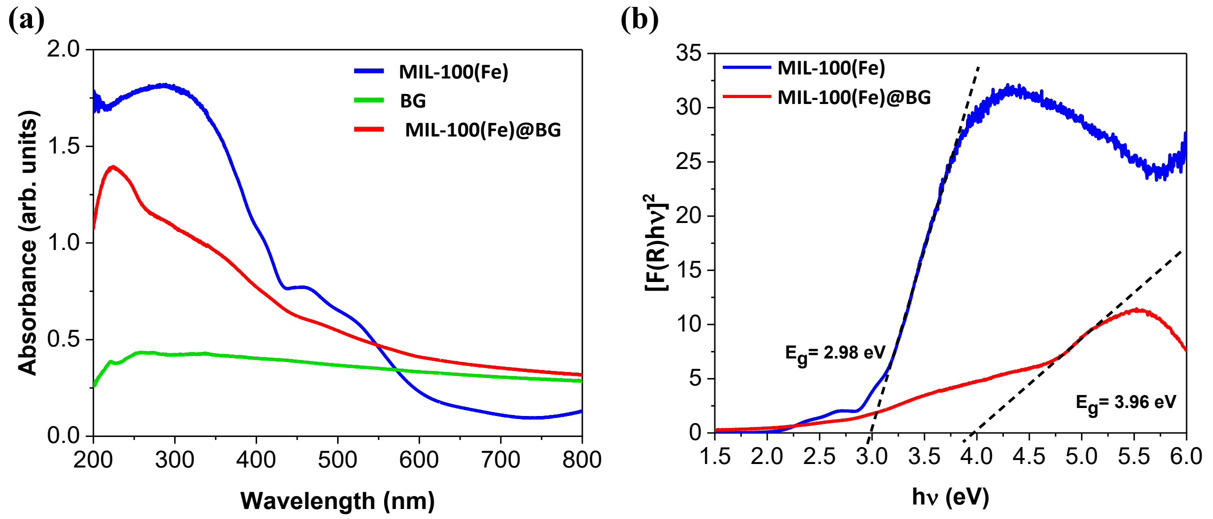

Figure S4. UV-Vis diffuse reflectance spectra of MIL-100(Fe)@BG, MIL-100, and BG (a); Tauc plots (b) illustrating the band gap estimation for MIL-100(Fe) and MIL-100(Fe)@BG.

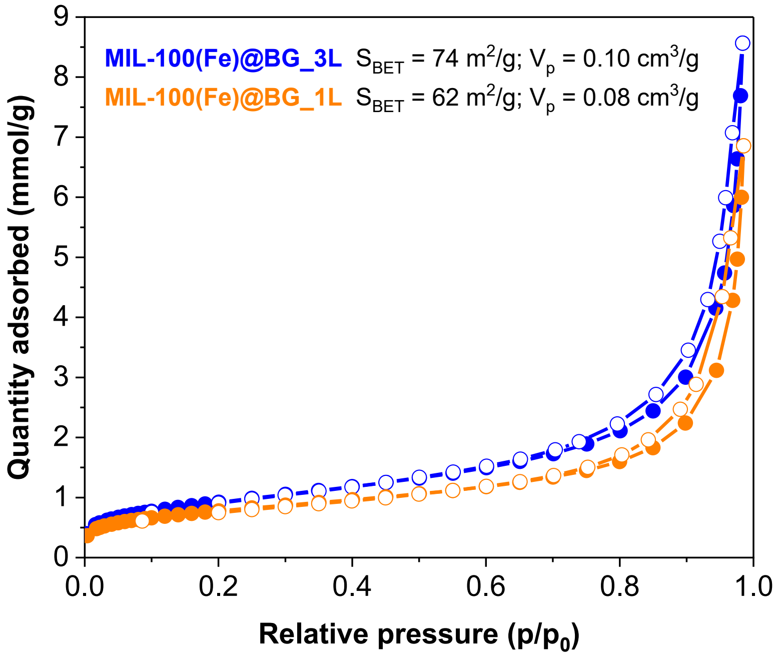

Figure S5. N_2 adsorption (filled symbols) and desorption (empty symbols) isotherms of MIL-100(Fe)@BG prepared using one and three deposition layers, together with the corresponding BET surface area and pore volume values. For MIL-100(Fe)@BG obtained after five deposition cycles, the reader is referred to Figure 1d in the main manuscript.

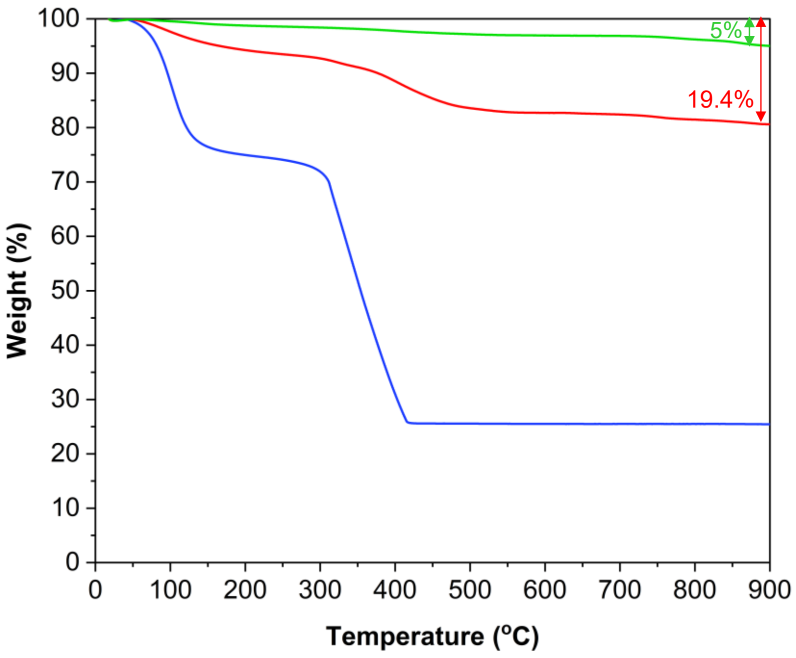

Figure S6. TG curves of BG (green), MIL-100(Fe)@BG (red), and MIL-100(Fe) (blue).

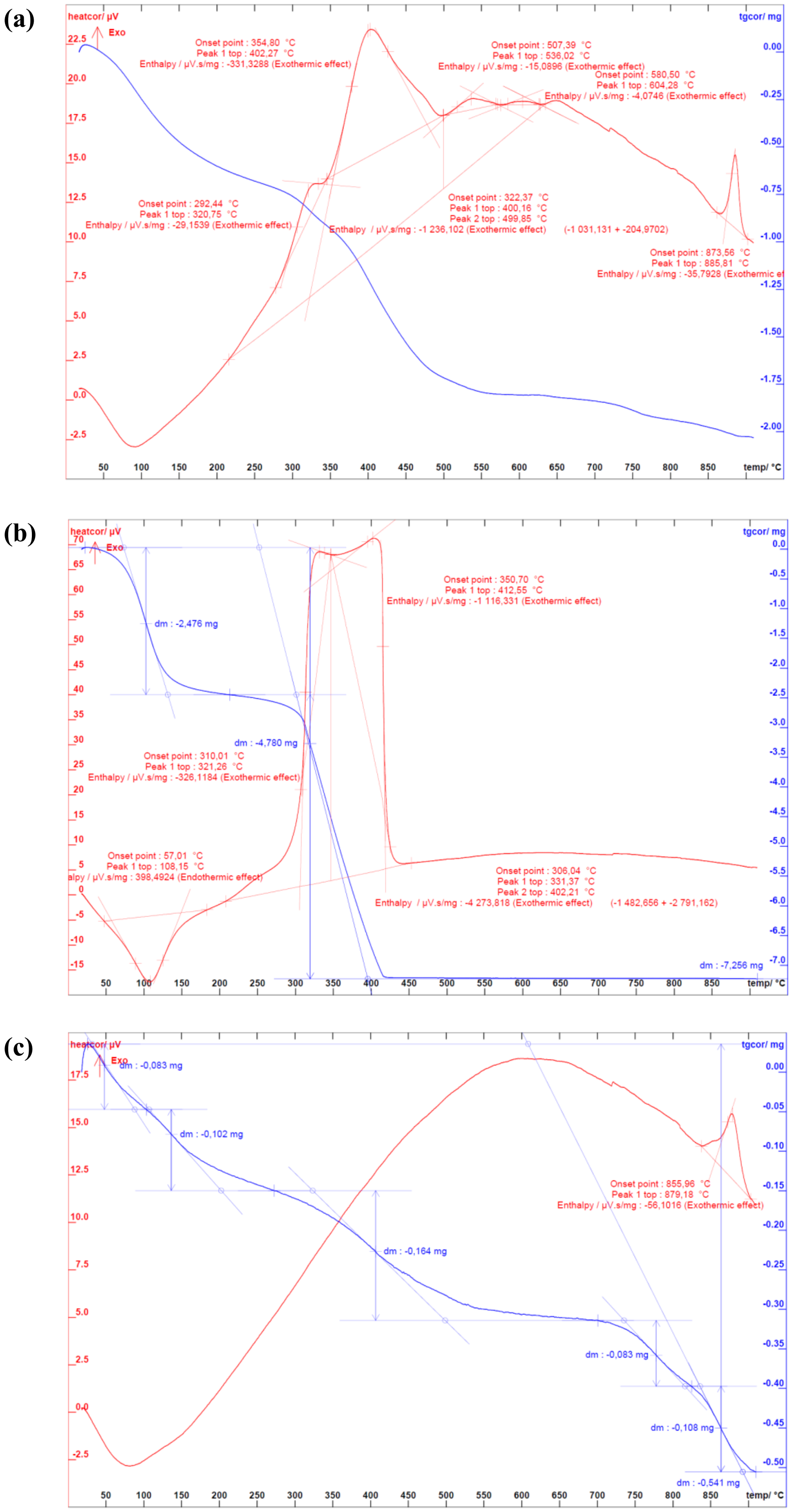

Figure S7. TG-DTA curves of MIL-100(Fe)@BG (a), MIL-100(Fe) (b) and BG (c).

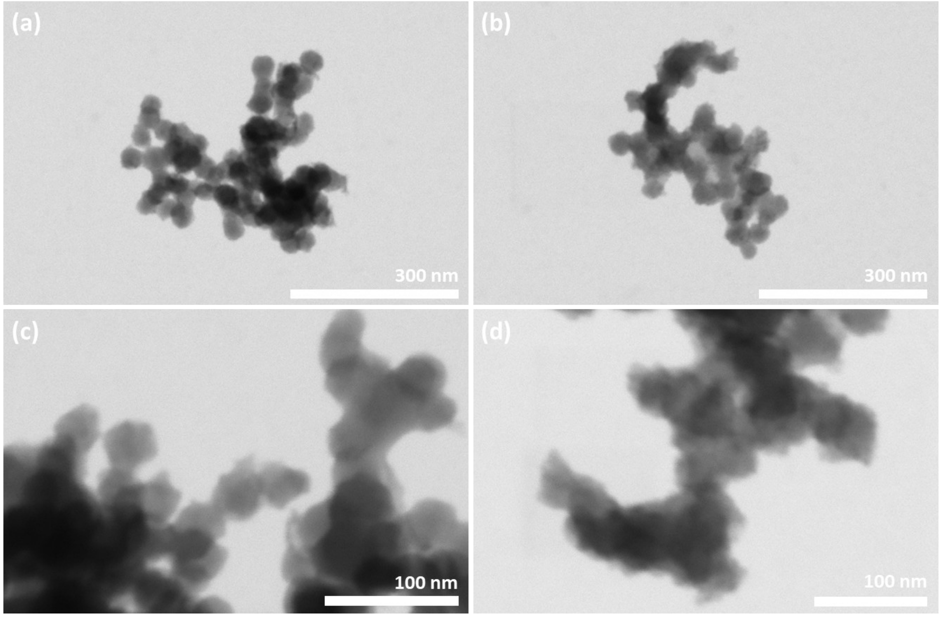

Figure S8. STEM images of MIL-100(Fe)@BG prepared using one deposition layer (a, c) and three deposition layers (b, d) of MIL-100(Fe).

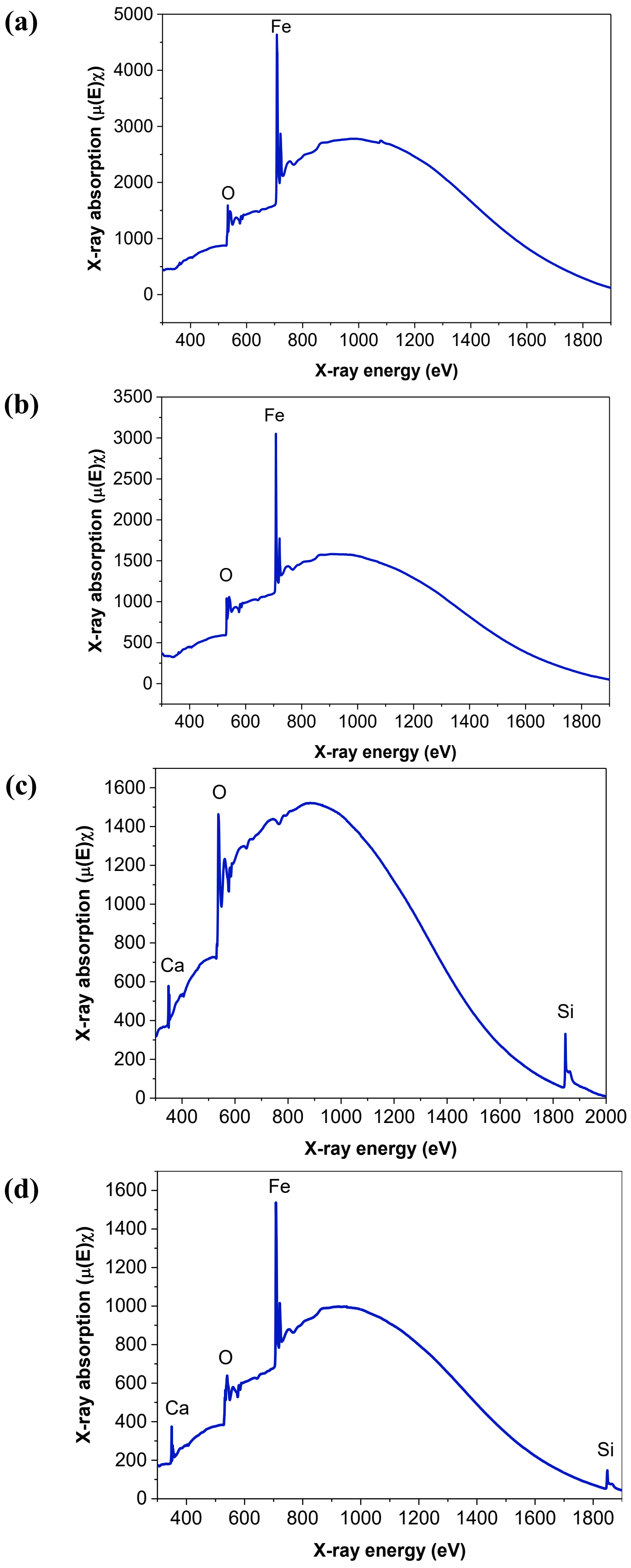

Figure S9. XAS spectra of Fe-cluster (a), MIL-100(Fe) (b), BG (c), and MIL-100(Fe)@BG (d).

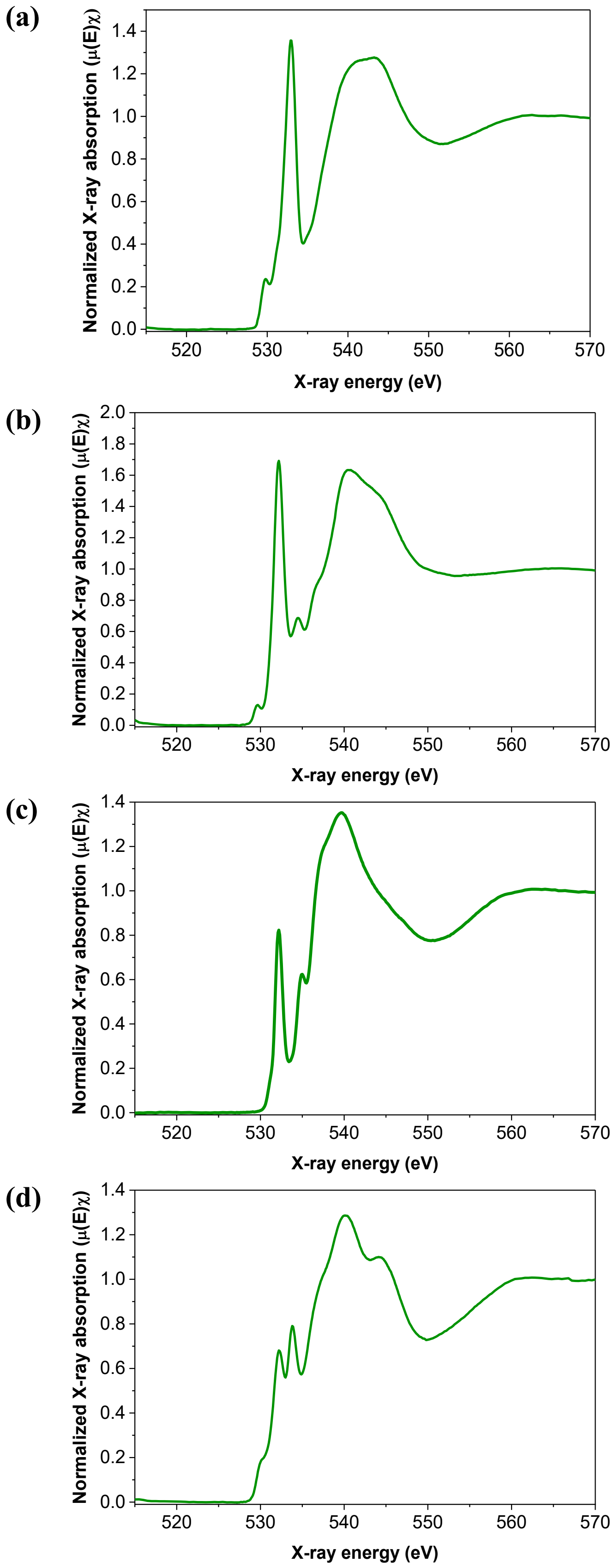

Figure S10. Normalized O K-edge XAS spectra of Fe-cluster (a), MIL-100(Fe) (b), BG (c), and MIL-100(Fe)@BG (d).

(a)

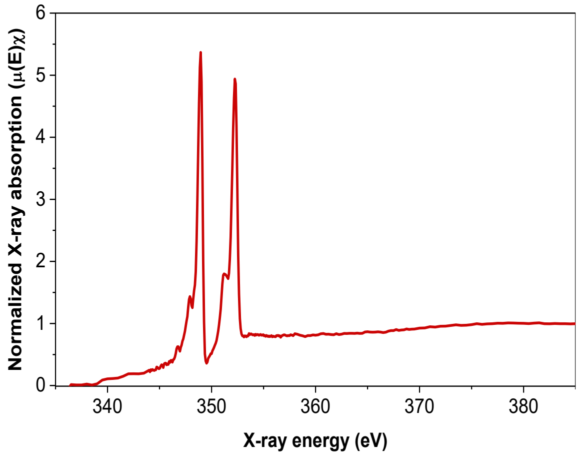

(b)

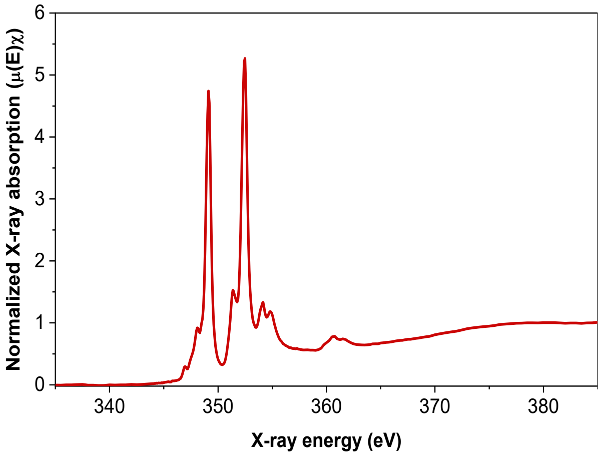

Figure S11. Normalized Ca L-edge XAS spectra of BG (a) and MIL-100(Fe)@BG (b).

(a)

(b)

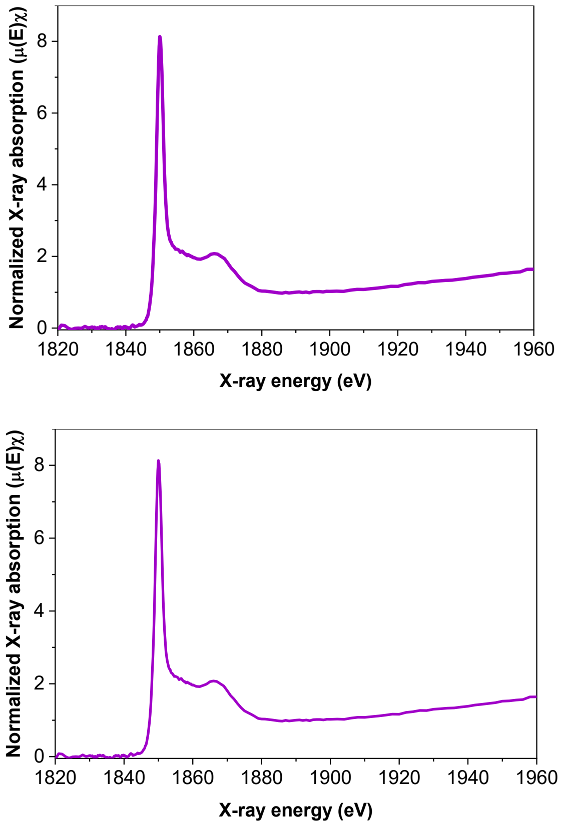

Figure S12. Normalized Si K-edge XAS spectra of BG (a) and MIL-100(Fe)@BG (b).

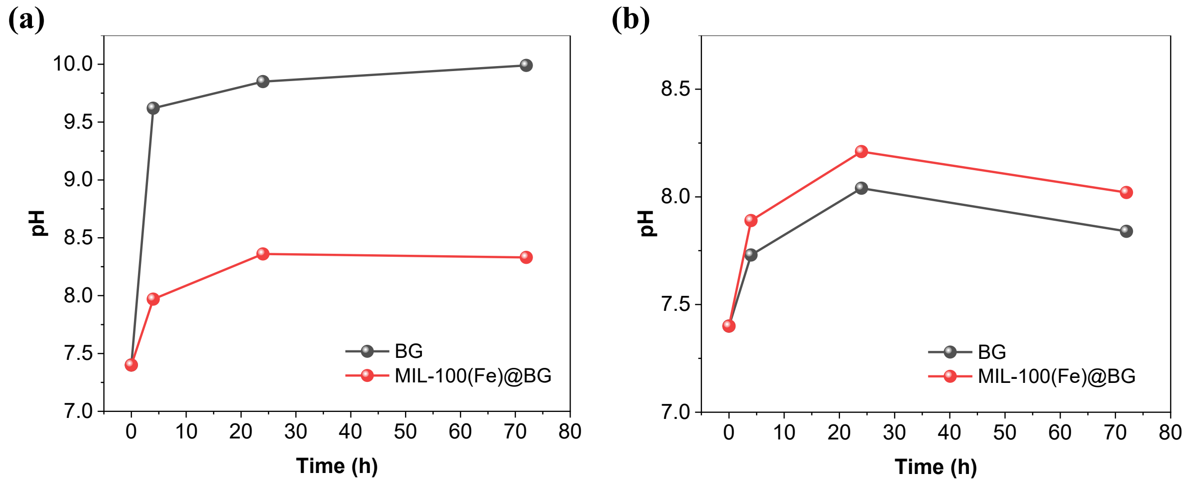

Figure S13. pH changes in DPBS (a) and SBF (b) for BG and MIL-100(Fe)@BG measured after 4, 24, and 72 hours (prior to the first medium exchange).

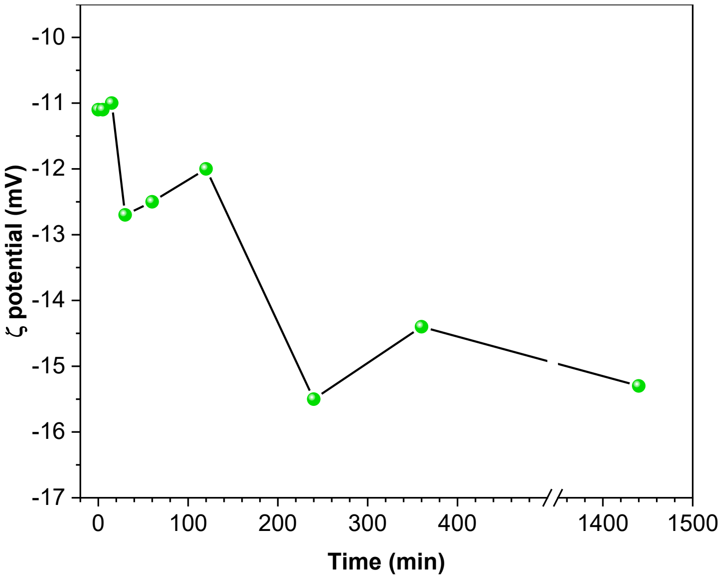

Figure S14. ζ-potential of MIL-100(Fe)@BG at 37 °C in DPBS. For clarity, error bars have been omitted in the plot; full numerical data and standard deviations are provided in Table S2.

Table S2. ζ-potential (mV) of MIL-100(Fe)@BG nanoparticles (1.5 mg/mL) incubated in DPBS (pH 7.4) at 37 °C and measured at selected time points.

Time	ζ potential (mV)
0	-11.1 ± 1.1
5 min	-11.1 ± 0.8
15 min	-11.0 ± 0.8
30 min	-12.7 ± 1.0
1 h	-12.5 ± 0.8
2 h	-12.0 ± 1.0
4 h	-15.5 ± 0.9
6 h	-14.4 ± 1.1
24 h	-15.3 ± 0.8

(a)

(b)

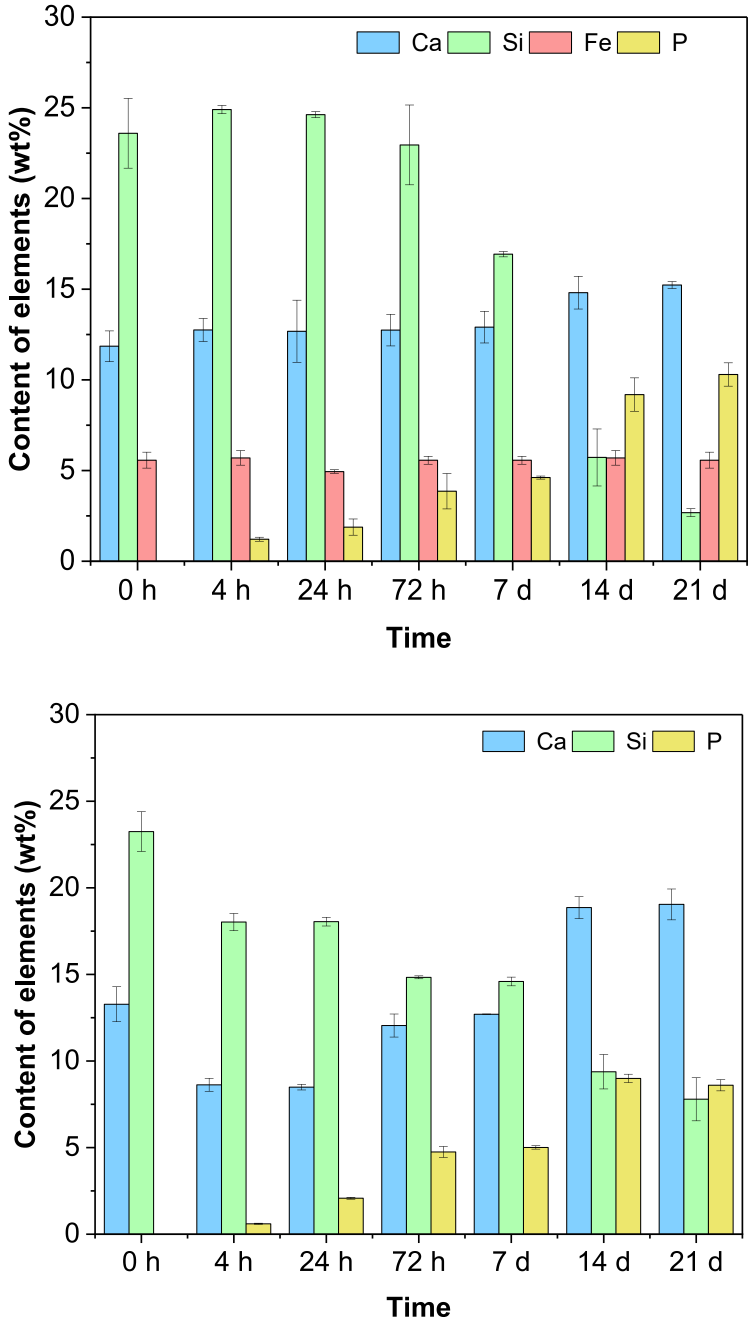

Figure S15. Analysis of the composition of MIL-100(Fe)@BG (a) and BG (b) during bioactivity testing in SBF, monitored by ICP-OES (37 ºC, 4 hours - 21 days).

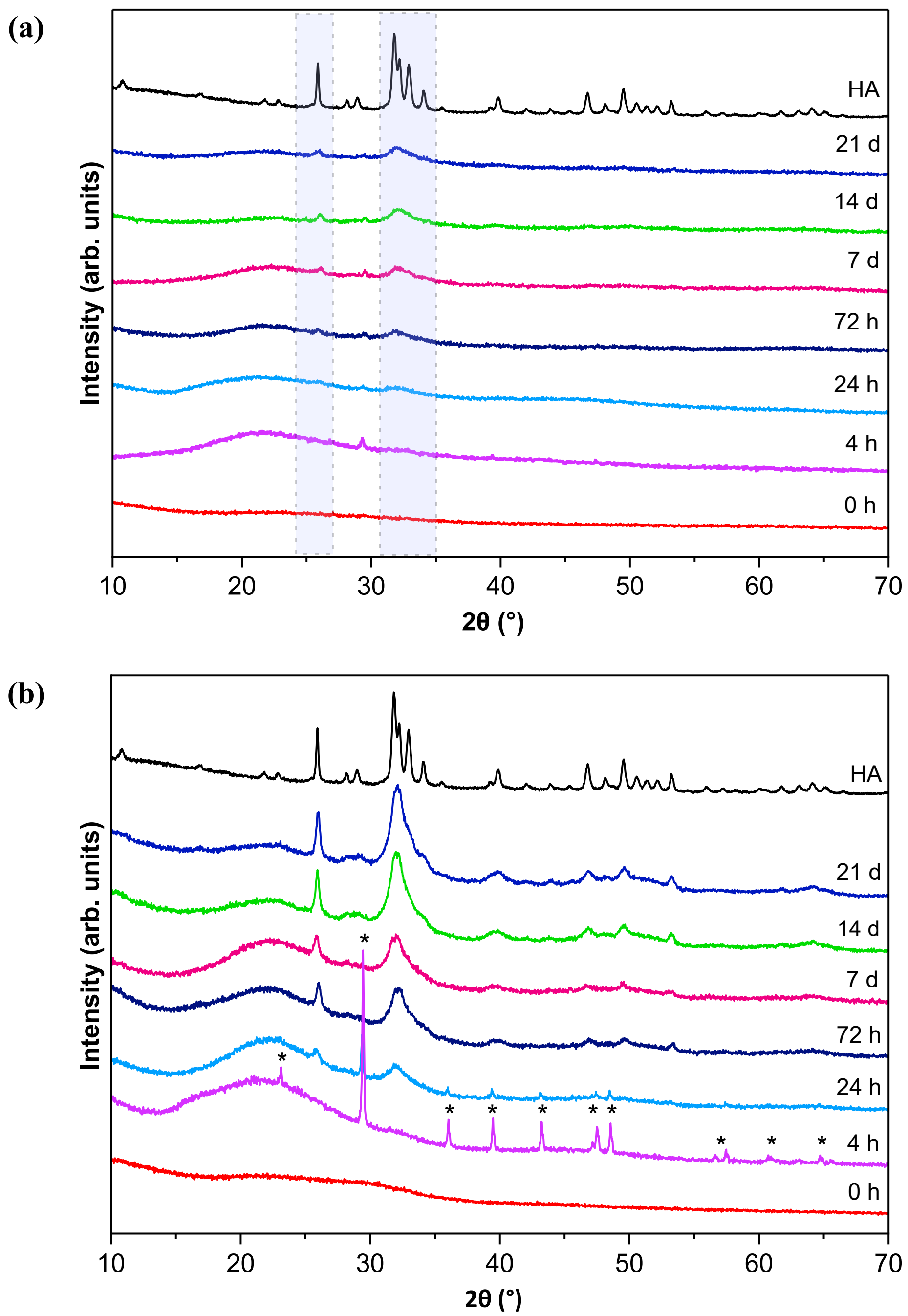

Figure S16. PXRD patterns of MIL-100(Fe)@BG (a) and BG (b) after immersion in SBF (37 ºC, 4 hours - 21 days). Diffraction peaks specific to calcium carbonate are marked with an asterisk.

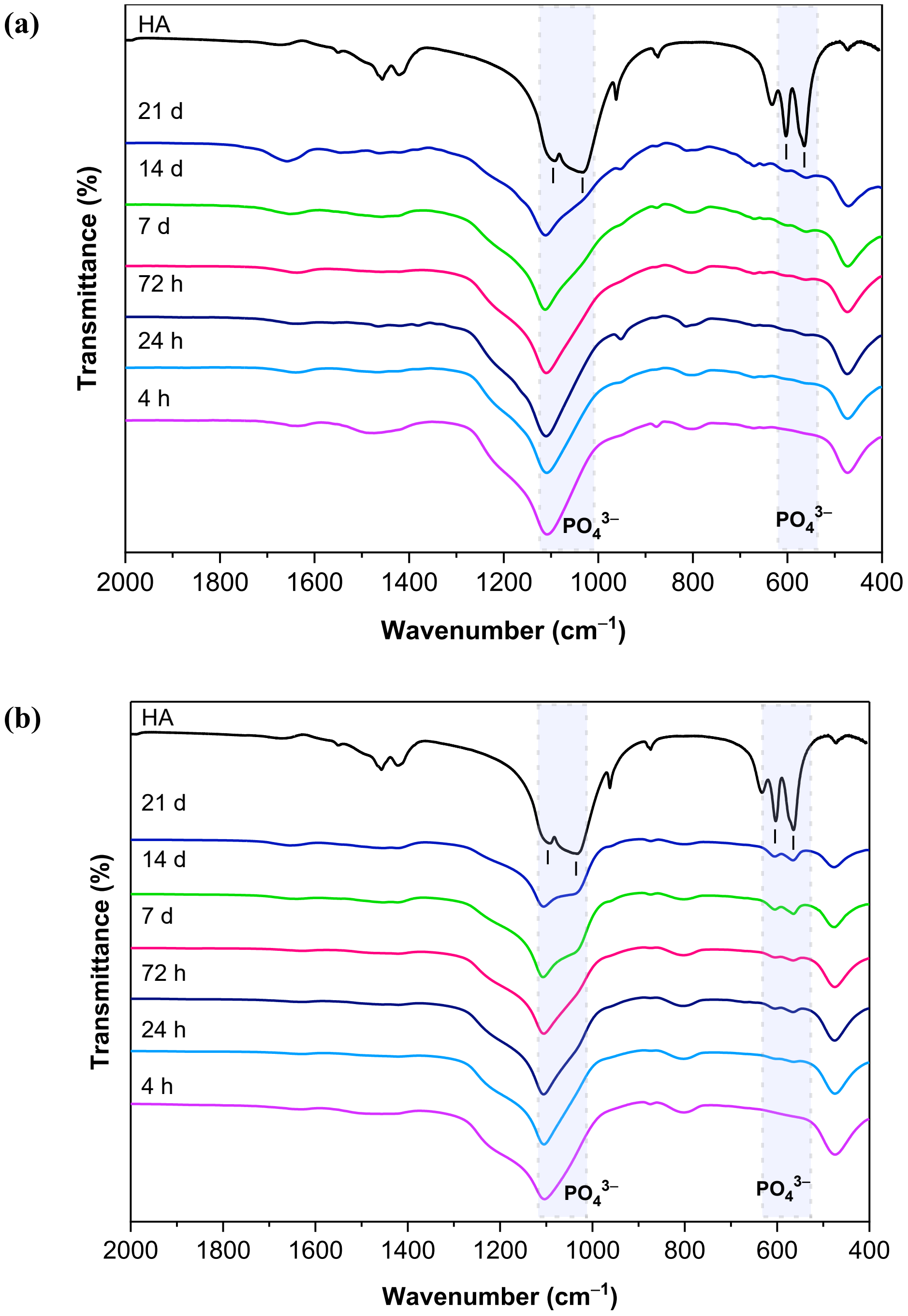

Figure S17. FTIR spectra of MIL-100(Fe)@BG (a) and BG (b) after immersion in SBF (37 ºC, 4 hours - 21 days).

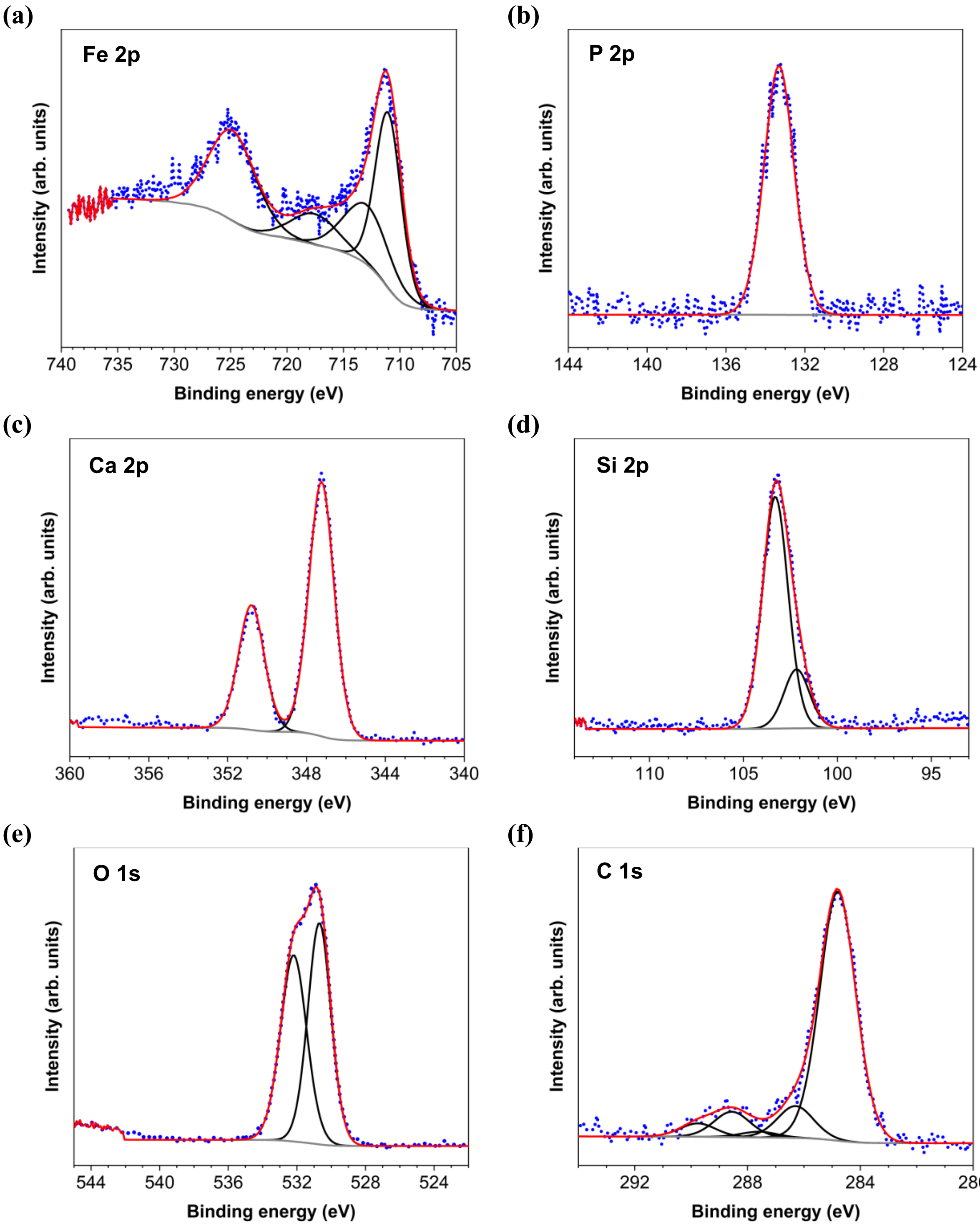

Figure S18. High-resolution XPS spectra of MIL-100(Fe)@BG after 7 days of immersion in DPBS (37 ºC, pH 7.4): Fe 2p (a), P 2p (b), Ca 2p (c), Si 2p (d), O 1s (e), and C 1s (f).

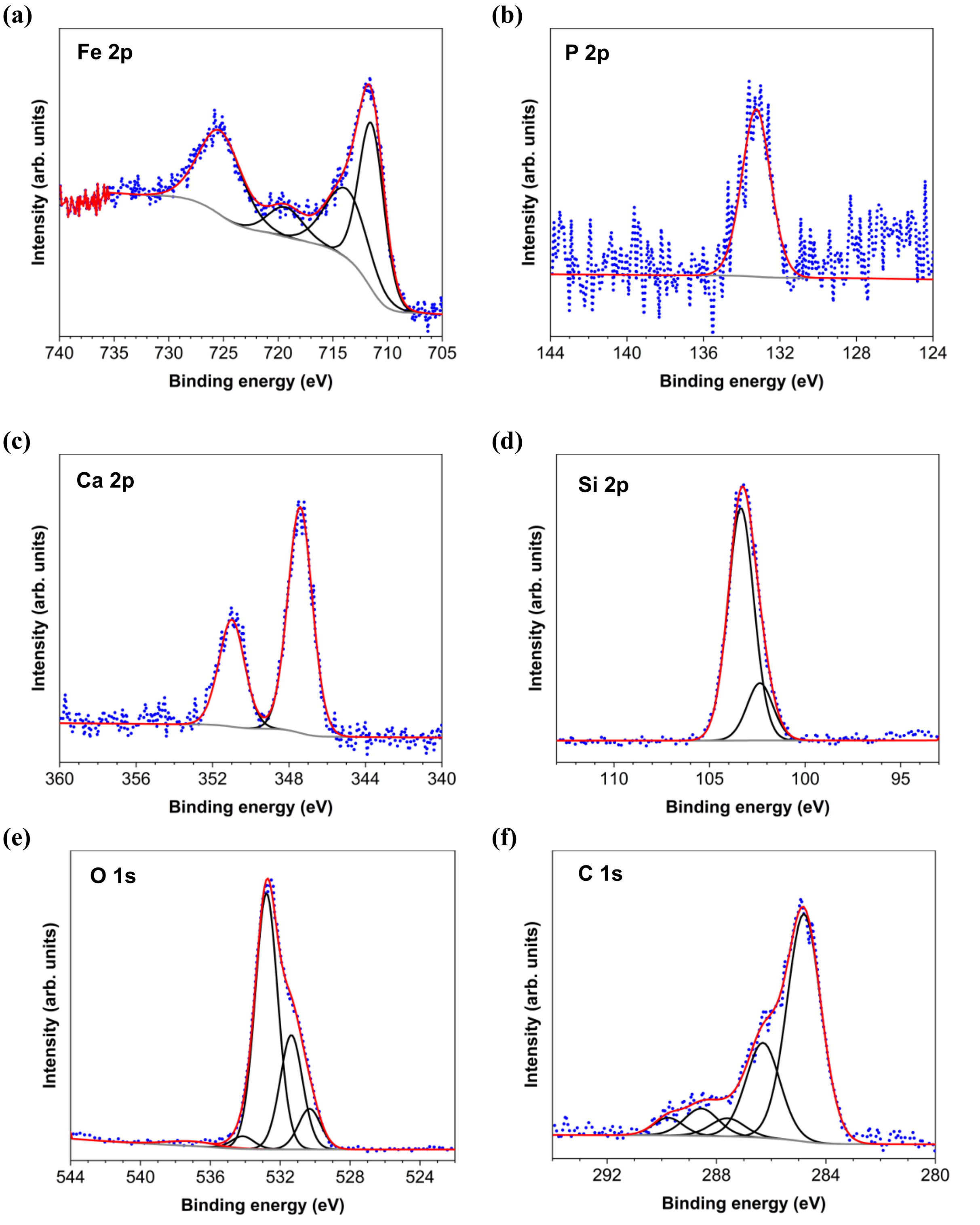

Figure S19. High-resolution XPS spectra of MIL-100(Fe)@BG after 7 days of immersion in SBF (37 ºC, pH 7.4): Fe 2p (a), P 2p (b), Ca 2p (c), Si 2p (d), O 1s (e), and C 1s (f).

Table S3. XPS fitting results showing binding energy values and corresponding chemical assignments for key elements in MIL-100(Fe)@BG incubated for 7 days.

Peak	MIL-100(Fe)@BG_DPBS		MIL-100(Fe)@BG_SBF		Assignment	Reference
	Binding energy (eV)	Area (%)	Binding energy (eV)	Area (%)		
Fe 2p	711.1	35.2	711.6	35.4	Fe(III) in $2p_{3/2}$	[1]
	712.9	21.0	713.8	24.8	Fe(III) in $2p_{3/2}$	
	717.6	11.1	719.3	8.9	Satellite	
	724.8	32.7	725.2	30.9	Fe(III) in $2p_{1/2}$	
Ca 2p	347.2	50.7	347.4	50.7	Ca(II) in $2p_{3/2}$	[2], [9]
	350.8	49.3	351.0	49.3	Ca(II) in $2p_{1/2}$	
P 2p	133.3	100.0	133.2	100.0	P-O	[9]
Si 2p	103.3	79.7	103.4	80.2	Si–O–Si	[3]
	102.2	20.3	102.4	19.8	Si–O–Ca	[4]
O 1s	530.7	51.3	530.3	9.6	O–Fe / O–P / O–C	[1], [10]
			531.4	27.0	Si–O–Ca / R–O–C=**O**	[1], [4-5]
	532.2	48.7	532.8	60.4	C–OH / Si–O–Si / Ca–OH	[1], [4-5], [10]
			534.1	3.0	O=C–**O**–R / Si–OH	[1], [5]
C 1s	284.8	76.6	284.8	59.3	C=C / C–C/ C–H	[1], [6]
	286.3	9.8	286.3	24.8	C–O	[6]
	287.6	1.8	287.6	4.7	C=O	[7]
	288.6	7.8	288.6	7.1	R–O–C=O	[1]
	289.8	4.1	289.8	4.0	$\pi \rightarrow \pi^*$/ CO_3^{2-}	[1], [8]

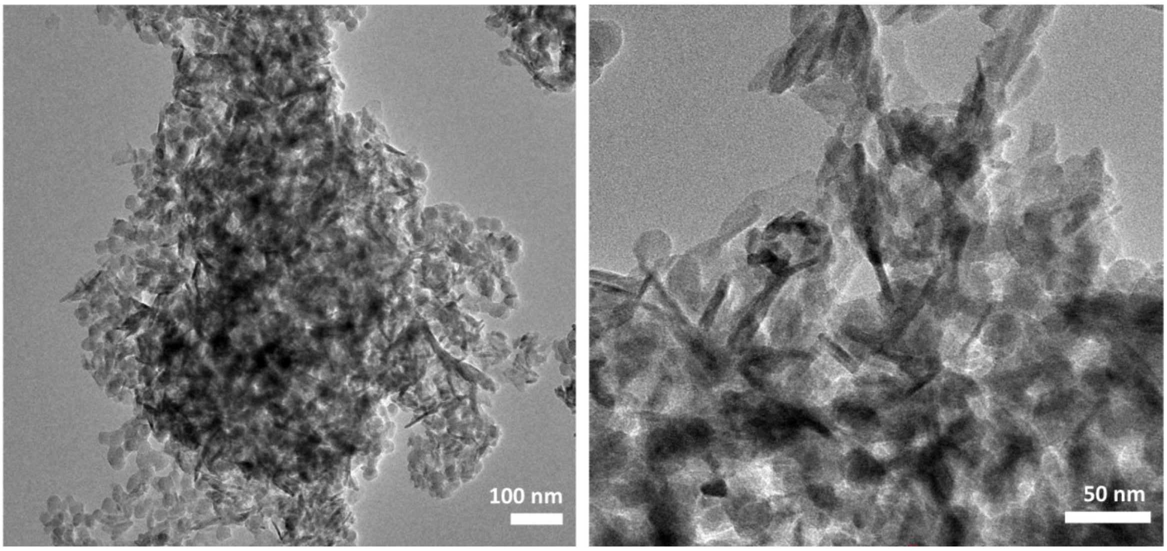

Figure S20. TEM images of BG after 21 days of immersion in DPBS (37 ºC, pH 7.4).

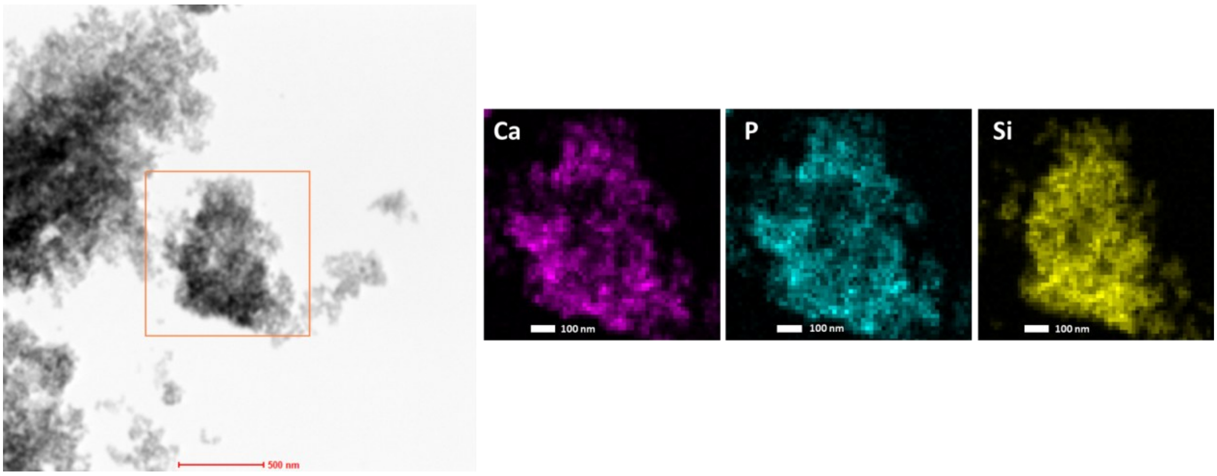

Figure S21. STEM image and EDS mapping analysis of BG after 21 days of immersion in DPBS (37 ºC, pH 7.4).

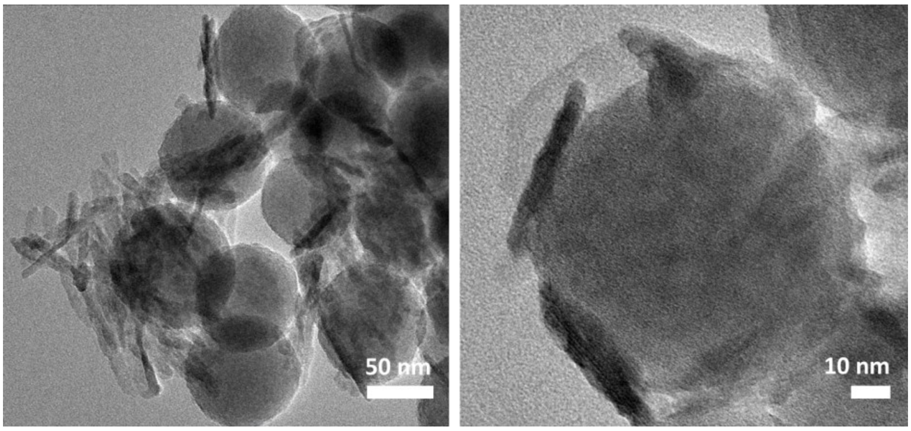

Figure S22. TEM images of BG after 21 days of immersion in SBF (37 ºC, pH 7.4).

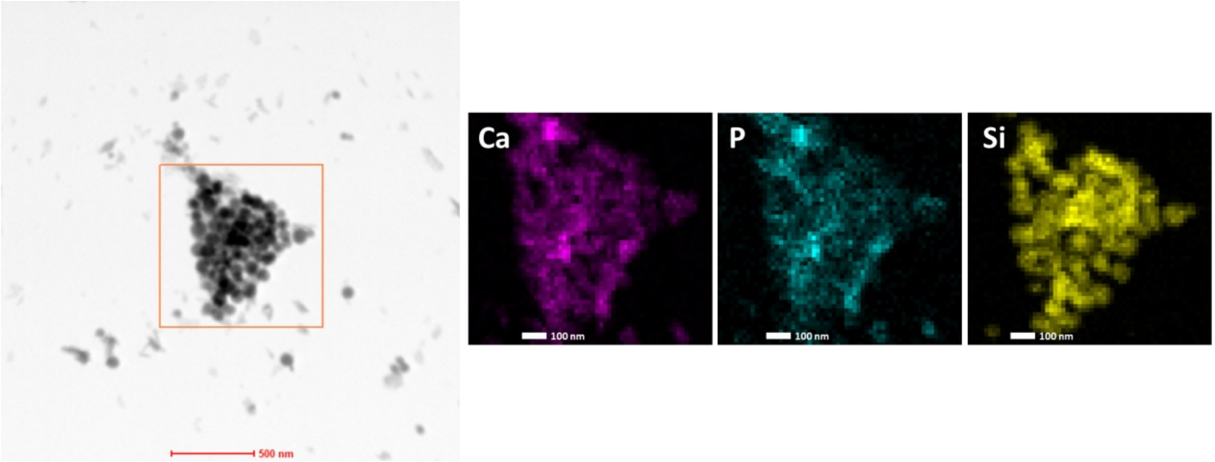

Figure S23. STEM image and EDS elemental mapping analysis of BG after 21 days of immersion in SBF (37 ºC, pH 7.4).

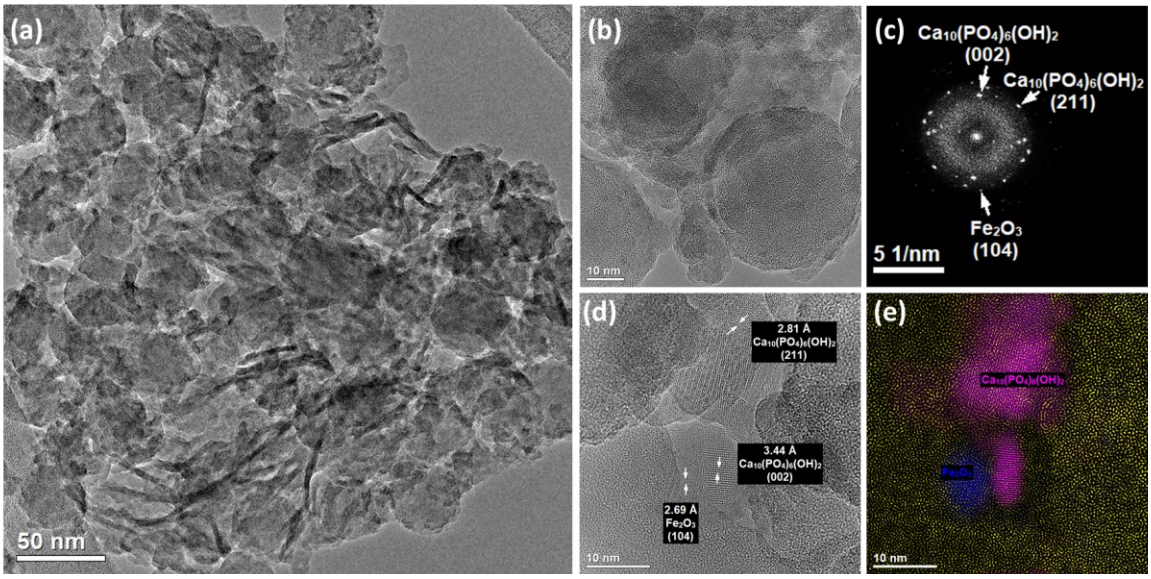

Figure S24. HRTEM images (a-b) with FFT and phase analysis (c-e) of MIL-100(Fe)@BG after 21 days of immersion in SBF (37 ºC, pH 7.4).

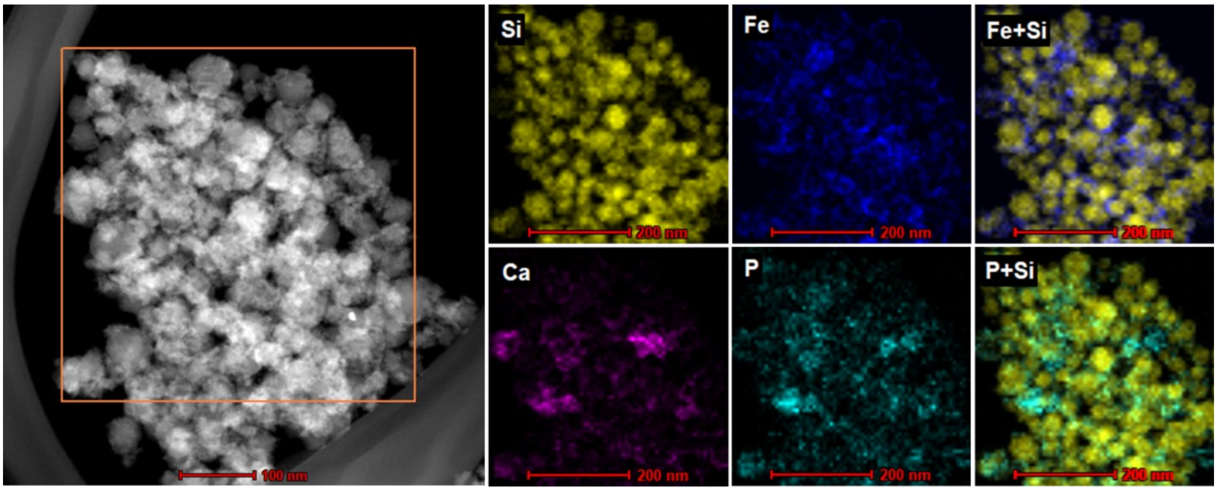

Figure S25. STEM image and EDS elemental mapping analysis of MIL-100(Fe)@BG after 21 days of immersion in SBF (37 ºC, pH 7.4).

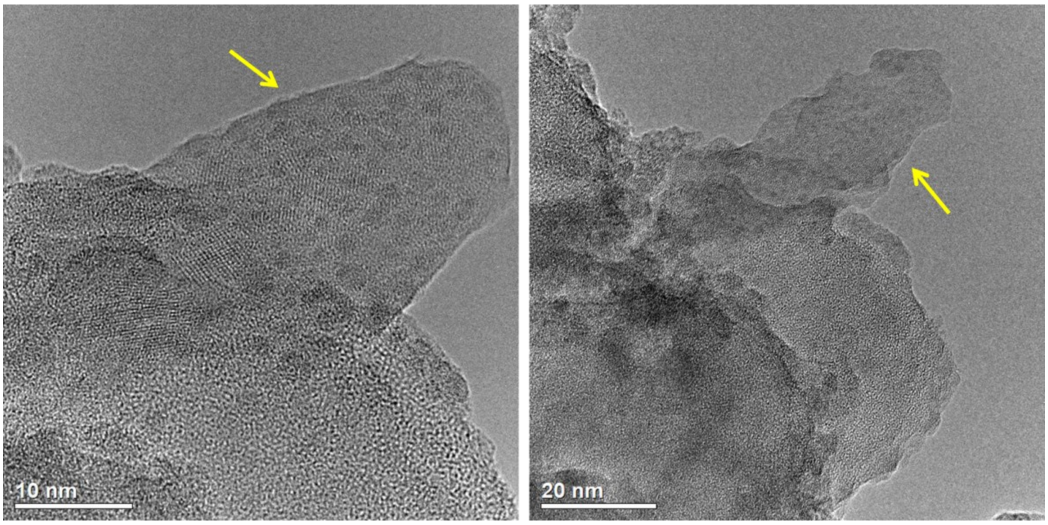

Figure S26. HRTEM images of MIL-100(Fe)@BG after 21 days of immersion in DPBS (37 ºC, pH 7.4) showing the formation of HA nanocrystallites with anchored Fe_2O_3 particles.

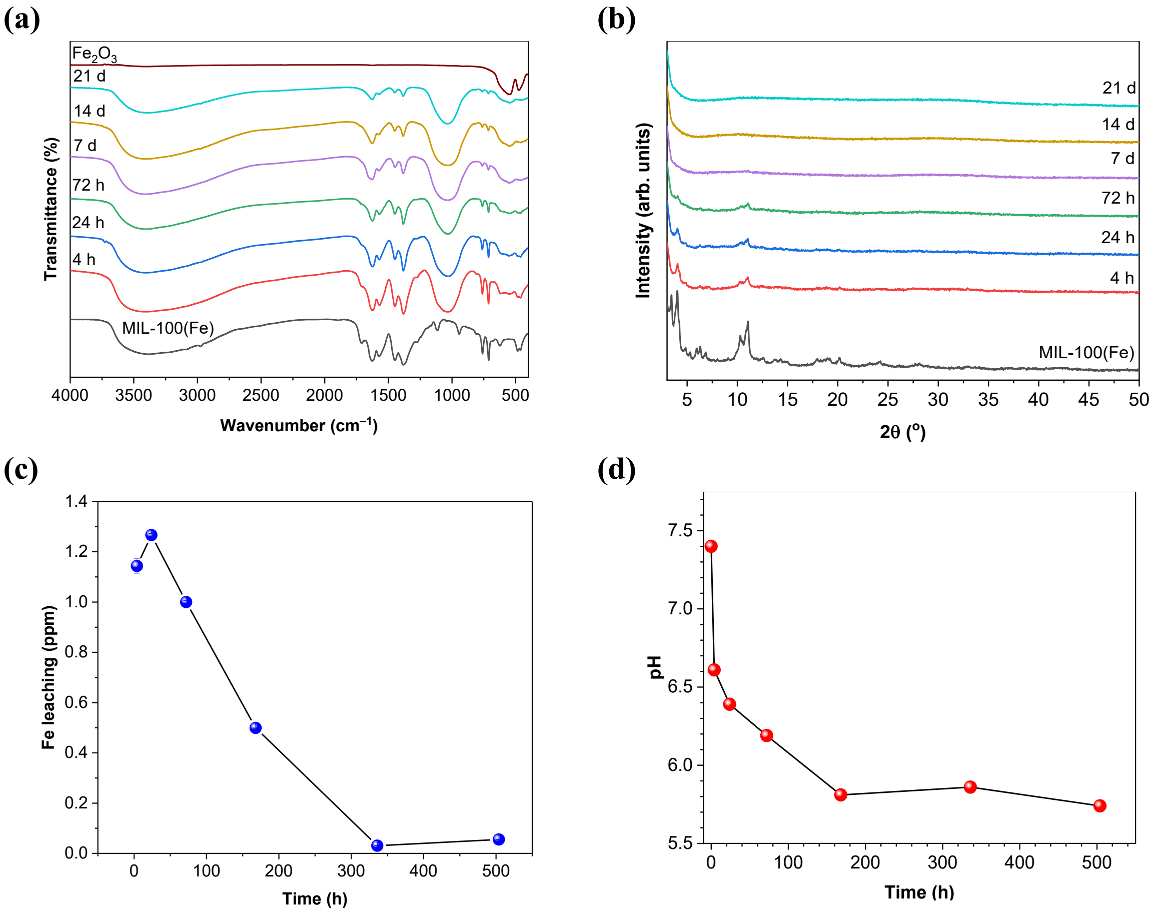

Figure S27. Stability assessment of MIL-100(Fe) under continuous incubation conditions (1.5 mg/mL, DPBS, 37 °C, pH 7.4; without medium refreshing): FTIR spectra (a), including a reference spectrum of commercial Fe_2O_3, PXRD patterns (b), Fe leaching determined by ICP-OES (c), and pH monitoring (d) after 4, 24, and 72 hours and 7, 14, and 21 days.

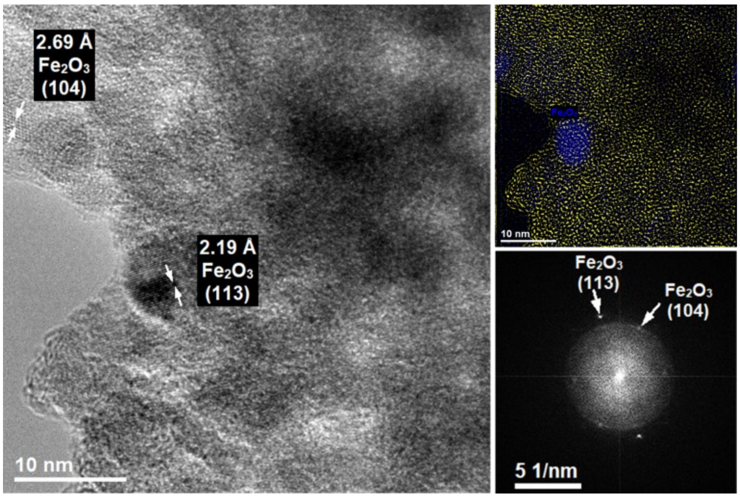

Figure S28. HRTEM image with FFT and phase analysis of MIL-100(Fe) after 21 days of incubation in DPBS (37 ºC, pH 7.4).

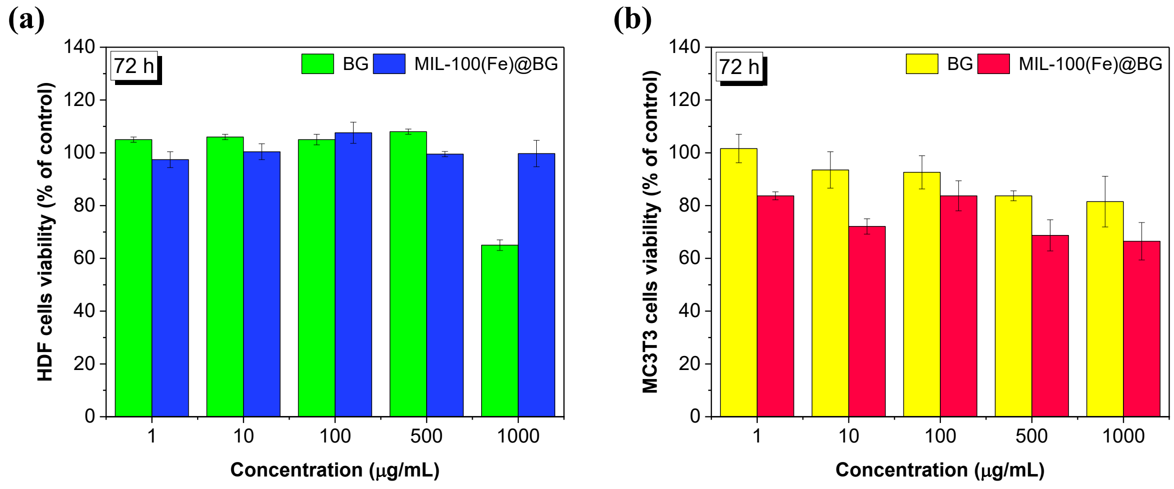

Figure S29. Viability of human dermal fibroblasts (HDF) (a) and mouse osteoblast precursors (MC3T3) (b) cultured with MIL-100(Fe)@BG and BG after 72 hours of incubation.

References

[1] Chávez, A. M.; Rey, A.; López, J.; Álvarez, P. M.; Beltrán, F. J. Critical Aspects of the Stability and Catalytic Activity of MIL-100(Fe) in Different Advanced Oxidation Processes. *Sep. Purif. Technol.* **2021**, *255*, 117660.

[2] Zhang, Y.; Zhang, J.; Jin, Y.; Zhang, J.; Hu, G.; Lin, S.; Yuan, R.; Liang, X.; Xiang, W. Construction and Nonlinear Optical Characterization of CuO Quantum Dots Doped Na_2O–CaO–B_2O_3–SiO_2 Bulk Glass. *J. Mater. Sci.: Mater. Electron.* **2017**, *28*, 13201–13208.

[3] Barrioni, B. R.; Norris, E.; Jones, J. R.; Pereira, M. M. The Influence of Cobalt Incorporation and Cobalt Precursor Selection on the Structure and Bioactivity of Sol–Gel-Derived Bioactive Glass. *J. Sol-Gel Sci. Technol.* **2018**, *88*, 309–321.

[4] Serra, J.; González, P.; Liste, S.; Serra, C.; Chiussi, S.; León, B.; Hupa, M. FTIR and XPS studies of bioactive silica based glasses. *J. Non-Cryst. Solids* **2003**, *332*, 20– 27.

[5] Zhang, Y.; Zhu, L.; Chen, L.; Liu, L.; Ye, G. Influence of Magnesia on Demoulding Strength of Colloidal Silica-Bonded Castables. *Rev. Adv. Mater. Sci.* **2019**, *58*, 32–37.

[6] Flores, C. V.; Obeso, J. L.; Viltres, H.; Peralta, R. A.; Ibarra, I. A.; Leyva, C. Efficient and effective removal of toluene from aqueous solution using MIL-100(Fe). *Environ. Sci.: Water Res. Technol.* **2024**, *10*, 2142–2147.

[7] Biesinger, M. C. Accessing the Robustness of Adventitious Carbon for Charge Referencing (Correction) Purposes in XPS Analysis: Insights from a Multi-User Facility Data Review. *Appl. Surf. Sci.* **2022**, *597*, 153681.

[8] Fournier, V.; Marcus, P.; Olefjord, I. Oxidation of Magnesium. *Surf. Interface Anal.* **2002**, *34*, 494–497.

[9] Hanawa, T.; Ota, M. Calcium Phosphate Naturally Formed on Titanium in Electrolyte Solution. *Biomaterials* **1991**, *12*, 767–774.

[10] Gomes, G. C.; Borghi, F. F.; Ospina, R. O.; López, E. O.; Borges, F. O.; Mello, A. Nd:YAG (532 nm) Pulsed Laser Deposition Produces Crystalline Hydroxyapatite Thin Coatings at Room Temperature. *Surf. Coat. Technol.* **2017**, *329*, 174–183.
